# A mass rearing cost calculator for the control of *Culex quinquefasciatus* in Hawaiʻi using the incompatible insect technique

**DOI:** 10.1186/s13071-022-05522-1

**Published:** 2022-12-05

**Authors:** Adam E. Vorsino, Zhiyong Xi

**Affiliations:** 1 Strategic Habitat Conservation Program, Ecological Services, Pacific Islands Fish and Wildlife Office, U.S. Fish and Wildlife Service, 300 Ala Moana Blvd Ste. 3-122, Honolulu, Hawaiʻi 96850 USA; 2 Department of Microbiology and Molecular Genetics, Michigan State University, 314 Giltner Hall, 293 Farm Lane, East Lansing, MI 48824 USA

**Keywords:** Sterile insect technique, Incompatible insect technique, Culicid, Infrastructure, Cost calculator, Hawaiʻi

## Abstract

**Background:**

Hawaiʻi’s native forest avifauna is experiencing drastic declines due to climate change-induced increases in temperature encroaching on their upper-elevation montane rainforest refugia. Higher temperatures support greater avian malaria infection rates due to greater densities of its primary vector, the southern house mosquito *Culex quinquefasciatus*, and enhance development of the avian malaria parasite *Plasmodium relictum*. Here we propose the use of the incompatible insect technique (IIT) or the combined IIT/sterile insect technique (SIT) for the landscape-scale (i.e., area-wide) control of *Cx. quinquefasciatus*, and have developed a calculator to estimate the costs of IIT and IIT/SIT applications at various sites in Hawaiʻi.

**Methods:**

The overall cost of the infrastructure, personnel, and space necessary to produce incompatible adult males for release is calculated in a unit of ~ 1 million culicid larvae/week. We assessed the rearing costs and need for effective control at various elevations in Hawaiʻi using a 10:1 overflooding ratio at each elevation. The calculator uses a rate describing the number of culicids needed to control wild-type mosquitoes at each site/elevation, in relation to the number of larval rearing units. This rate is a constant from which other costs are quantified. With minor modifications, the calculator described here can be applied to other areas, mosquito species, and similar techniques. To test the robustness of our calculator, the Kauaʻi-specific culicid IIT/SIT infrastructure costs were also compared to costs from Singapore, Mexico, and China using the yearly cost of control per hectare, and purchasing power parity between sites for the cost of 1000 IIT/SIT males.

**Results:**

As a proof of concept, we have used the calculator to estimate rearing infrastructure costs for an application of IIT in the Alakaʻi Wilderness Reserve on the island of Kauaʻi. Our analysis estimated an initial investment of at least ~ $1.16M with subsequent yearly costs of approximately $376K. Projections of rearing costs for control at lower elevations are ~ 100 times greater than in upper elevation forest bird refugia. These results are relatively comparable to those real-world cost estimates developed for IIT/SIT culicid male production in other countries when inflation and purchasing power parity are considered. We also present supplemental examples of infrastructure costs needed to control *Cx. quinquefasciatus* in the home range of ʻiʻiwi *Drepanis coccinea*, and the yellow fever vector *Aedes aegypti*.

**Conclusions:**

Our cost calculator can be used to effectively estimate the mass rearing cost of an IIT/SIT program. Therefore, the linear relationship of rearing infrastructure to costs used in this calculator is useful for developing a conservative cost estimate for IIT/SIT culicid mass rearing infrastructure. These mass rearing cost estimates vary based on the density of the targeted organism at the application site.

**Graphic Abstract:**

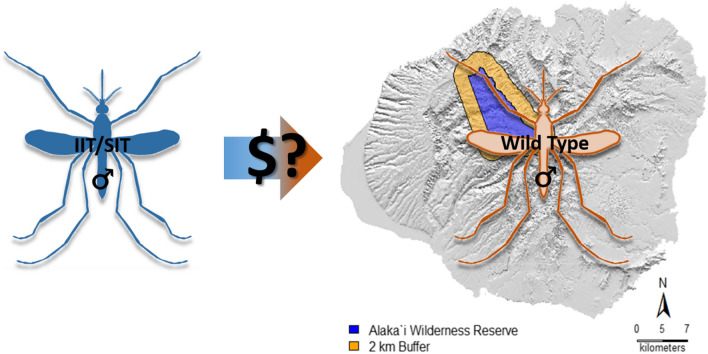

**Supplementary Information:**

The online version contains supplementary material available at 10.1186/s13071-022-05522-1.

## Background

Hawaiʻi’s honeycreepers (Drepanididae) are iconic, culturally significant native forest birds relegated to small remnant populations in the upper elevation of montane rainforests [1, 2]. These native forest bird refugia will likely disappear due to increasing temperatures [3, 4]. However, because disease inhibition at lower temperatures maintains these refugia, its disappearance does not necessarily mean the complete loss of forest bird habitat due to climate change [2]. Current projections indicate that Hawaiʻi’s forest birds could maintain and possibly expand their populations in the absence of avian malaria (i.e., *Plasmodium relictum*) [4]. Avian malaria is generally absent from these upper elevation refugia due to the effect of temperature on its development, and the reproductive success of its primary vector, the southern house mosquito *Culex quinquefasciatus* (Diptera: Culicidae). As temperature affects the development of *P. relictum* and *Cx. quinquefasciatus,* it has been used to infer prevalence at various sites [5]. Current models project that as the temperature in these refugia climb, *Cx. quinquefasciatus* and *P. relictum* will successfully develop, spread, and thrive [5]. Culicid collections in and around these refugia corroborate those projections (C. Crampton, pers. comm.). If mosquito control tools that can be broadcasted and distributed across a broad swath of ecologically and topographically complex landscapes (hereafter: landscape-scale) are not applied quickly, efficiently, and effectively, Hawaiʻi will continue to see its native avifauna drastically decline, leading to extinction events in the very near future [2, 4]. Though rearing honeycreepers in captivity to protect them from the disease threat of avian malaria (captive rearing) is possible, it is at best a backstop until the threat to the species is removed. Therefore, it has become obvious that to stop the extinction of these iconic species we must quickly develop and apply an efficient and effective tool for landscape-scale control of *Cx. quinquefasciatus*.

While both male and female *Cx. quinquefasciatus* take sugar meals from plants, only females, prior to laying an egg raft (consisting of ~ 200 eggs), must take a blood meal. The gonotrophic cycle is a female-specific life history trait describing the interval between taking a blood meal and egg laying. Female *Cx. quinquefasciatus* can complete multiple gonotrophic cycles in a lifetime, but mate only once [6]. Area-wide mosquito control tools take advantage of these culicid life history characteristics and develop males that cannot viably produce offspring with a targeted population of interest. Two such tools explored here are the incompatible insect technique (IIT) and the sterile insect technique (SIT) [7–9]. The IIT uses a mechanism referred to as cytoplasmic incompatibility (CI), which is the process of embryonic cell death that occurs when a *Wolbachia*-infected male mates with an uninfected female, or a female that carries a different strain of *Wolbachia* [9]. This is in contrast to SIT in which radiation induces sterility in males [8]. Field trials of IIT have resulted in successfully eradicating *Cx. quinquefasciatus* [10] and the near-elimination of the Asian tiger mosquito *Aedes albopictus* and the yellow fever mosquito *Aedes aegypti* populations [11–13]. Area-wide implementation of SIT has successfully suppressed populations of screwworm, medfly, and tsetse flies [8, 14], with recent encouraging progress in field trials for *Aedes spp.* control [15, 16].

There are two main limitations associated with the SIT and IIT approaches. For SIT, although recent efforts have improved the mating competitiveness of irradiated males, in general radiation has detrimental effects on the fitness of fragile male culicids [17]. The limitation associated with IIT is the unintentional release of *Wolbachia*-infected females into the field, as there is currently no error-free sex separation approach. Imperfect sex separation may result in the failure to suppress a targeted population because mating will be compatible if both males and females carry the same strain of *Wolbachia*; therefore, release of a different *Wolbachia* strain is needed for an equivalent level of suppression [18, 19]. To address the above issues a new approach was developed that combines IIT and SIT approaches to prevent the release of any residual fertile females [11]. The IIT/SIT approach is a cost-effective solution that will likely result in successful suppression of targeted mosquito populations [11, 20, 21]. As IIT, SIT, and IIT/SIT have been successfully developed and applied to control *Cx. quinquefasciatus* (and other culicids) in other tropical systems it is assumed that these strategies will be equivalently successful in Hawaiʻi [10, 18, 22–24].

Recently, a novel *Wolbachia* strain was successfully established in *Cx. quinquefasciatus* collected from Hawaiʻi, and complete CI was induced when these transinfected mosquitoes mated with their wild-type counterpart [25]. The next step for the application of this tool is to develop the infrastructure necessary to produce enough incompatible males for area-wide control of *Cx. quinquefasciatus*. Here we present a cost estimate calculator for both IIT and IIT/SIT mass rearing infrastructure adapted for Hawaiʻi from previous work on *Aedes* control [11, 26]. The output of the calculator is a table of costs for mosquito rearing using the IIT or the IIT/SIT approach [11, 23]. As a proof of concept, we have developed outputs for an area with the size of the Alakaʻi Wilderness Reserve, a known refugia of native avifauna on the island of Kauaʻi. These estimates of infrastructure cost are a critical step in the development of an effective integrated pest management (IPM) plan.

## Methods

### Review of variables used to estimate infrastructure costs

**Fig. 1:**
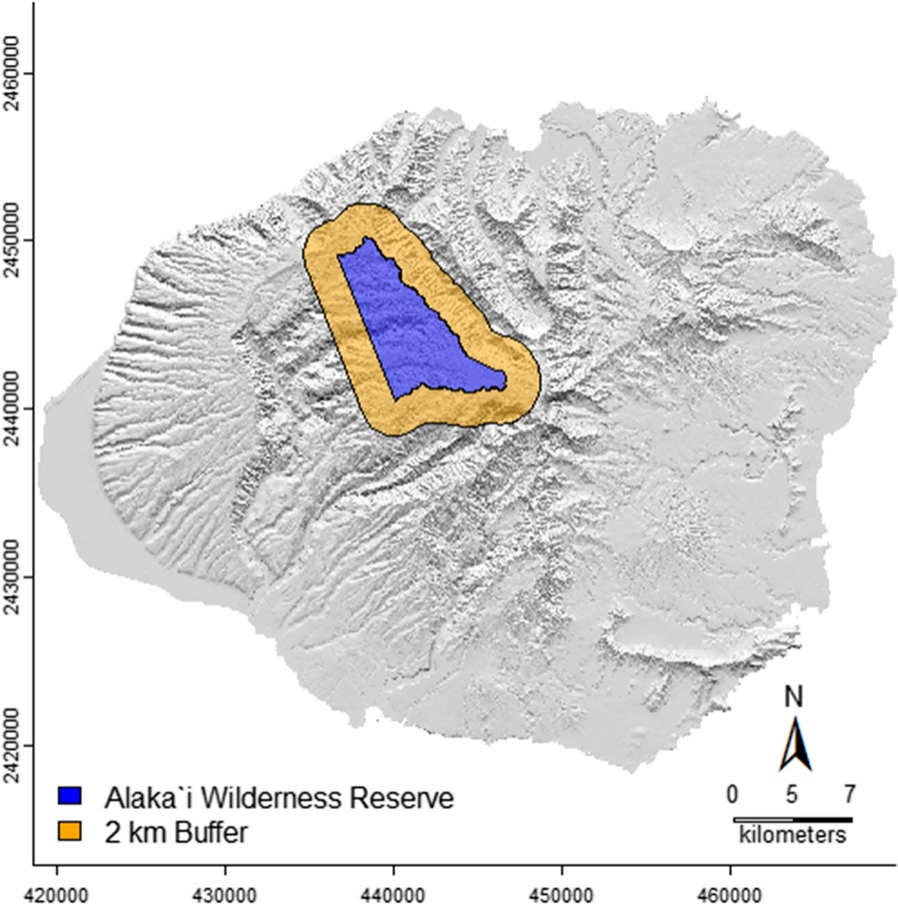
The theorized *Cx. quinquefasciatus* control area within and surrounding the Alakaʻi Wilderness Reserve on Kauaʻi (blue). The 2 km buffer surrounding the reserve (orange) represents the greatest cumulative mean distance of *Cx. quinquefasciatus* dispersal as defined by LaPointe [28]

Along with information related to the rearing of approximately 1.5 million IIT or IIT/SIT-derived male mosquitoes [11], approximations of Hawaiʻi-specific infrastructure, utility, and personnel costs used in this analysis were determined from utility, personnel costs, and indirect costs associated with other known Hawaiʻi facilities and organizations. The calculator presented here uses this compiled information to assess infrastructure costs (including rearing costs) associated with developing and running a *Cx. quinquefasciatus* rearing facility at a capacity needed to control known densities in an area the approximate size of the Alakaʻi Wilderness Reserve. The Alakaʻi Wilderness Reserve is within the Na Pali-Kona Forest Reserve, a reserve that includes known forest bird refugia, such as Kauaʻi’s Alakaʻi swamp (Fig. 1). We assume here that a 2 km^2^ buffer around the wilderness reserve (as derived from [27]) will minimize immigration of *Cx. quinquefasciatus* from outside source populations. This 2 km^2^ buffer represents the maximum mean distance traveled by *Cx. quinquefasciatus* [28]. Table 1 lists the variables used in the code to itemize the infrastructure costs and a description of each variable. Variables are partitioned into three main types in Table 1: those variables associated with ecology of Hawaiʻi and the culicids (mosquito and site information), those itemizing the basic infrastructure costs (basic infrastructure costs), and those associated with position and personnel expenditures (position and personnel costs). We developed this analysis in the R statistical environment [29] (Additional file 1: Code S1).

**Table 1:** Variable names and descriptions used in the R code to derive an estimate of infrastructure and personnel costs

Coded variable	Variable description
Mosquito and site information	
MaunaLoa.Mos.Sites	Sites from Samuel et al. [5] in which estimates of density were defined
MaunaLoa.Mos.Density.km	Mosquito densities (individuals/km^2^) at the various sites from Samuel et al. [5]
MaunaLoa.Elev.m	Approximate elevation of sites outlined in the *MaunaLoa.Mos.Sites* input in meters
Alakai.Area.ToCntrl.km	Approximate area of the Alakaʻi Wilderness Reserve with a 2 km buffer, in square kilometers
FemalePercent	The female percent of the sex ratio. A value of 50 indicates an equal sex ratio
Overflooding_Multiplier	Multiplier to derive the overflooding ratio needed for an effective control strategy. A value of 10 indicates that to suppress the wild-type population, a successful program needs 10 × the number of SIT/IIT-laboratory-derived culicids
Basic infrastructure costs	
Year1.Only.Items	Infrastructure items needed in year 1 for rearing approximately 1.5 million IIT/SIT male culicids, not including personnel
Year1.Only.Costs	Approximate costs of *Year1.Only.Items* for rearing 1.5 million IIT/SIT male culicids
Electricity	Yearly electricity costs to rear approximately 1.5 million IIT/SIT males
LaboratorySpace	Cost of a laboratory space needed for rearing approximately 1.5 million IIT/SIT males
AllOtherYear.Items	Misc PCR/lab/field supplies for items used in each year of the control application (e.g., blood)
AllOtherYear.Costs	Approximate costs of *AllOtherYear.Items* for items used in each year of the control application
Position and personnel costs	
Personnel.Des	Types of technician positions to be funded
Wage.Mass.Rearing	Average hourly wage for the mass rearing technician position
Wage.Quality.Control	Average hourly wage for the quality control position
HoursPerYear	Yearly hours for each position
Fringe	Percent fringe costs for full-time employees

### Biological/ecological characteristics of *Cx. quinquefasciatus* used for the analysis

As noted in Table 1, mosquito densities were derived from [5] (see Appendix B Table 1B in [5]) and defined in that paper as the number of individuals per km^2^ for each site. Infrastructure costs were determined for all densities of *Cx. quinquefasciatus* at each elevation in which they were present, as it is assumed that *Cx. quinquefasciatus* densities vary based on temperature [5], and elevation can be used as an imperfect proxy for the variation in temperature between sites. Conducting the analysis such that it takes into account this variation in elevation allows the user to better estimate the effect of location and life history on cost.

The male-to-female sex ratio was maintained at 1:1 for most analyses conducted in this assessment. While maintaining all infrastructure, personnel and density costs, a comparison between an equal (1:1) and female-biased sex ratio (70% female, or 0.43:1 male-to-female ratio, the converse of that from [30]) was conducted to illustrate the change in cost associated with this variable. Sex ratios commonly seen in the literature for *Cx. quinquefasciatus* (or other culicids) vary [30–34], but are within the realm of both ratios used. Though female-biased sex ratios may be observed in the field due to the short longevity of males, in the lab production can be biased toward males, as male pupae tend to emerge first [30]. For a conservative estimate of costs we assume that the IIT male production colony is optimized to have an equal sex ratio.

### Infrastructure costs

As noted in the “Review of variables used to estimate infrastructure costs” section above, most first-year, and subsequent year, infrastructure costs were estimated using information published (as an average cost/km^2^) in [11]. An estimate of 1.5 million male culicids per week was initially used as a starting point, as it was thought to encompass our rearing needs (prior to this analysis); in this calculator, this constant is only used to incorporate sufficient rearing space, which is modified based on the densities of the treated area. Based on the previous experience in the setup of mass rearing facilities in Mexico and China, it was determined that a facility should be ~ 300–500 m^2^ (enough to produce 500k^–1^ million male SIT/IIT culicids) to be cost-effective, and allow for future scaling efforts. Therefore, it is conservatively assumed here that a 800 m^2^ Arthropod Containment Level 2 (ACL-2) facility (Table 2: *LaboratorySpace*) would be of sufficient size to rear approximately 1.5 million male *Cx. quinquefasciatus*, but this facility size could be optimized to rear double or triple that amount. All estimates of laboratory space size include space for larval and adult rearing and sorting. The cost of an 800 m^2^ facility was estimated using three converted (from ft^2^ to m^2^) median cost quotes (Additional file 3: Quotes S1), as defined per m^2^, and multiplied by the minimum size of a facility (800 m^2^). Also, modification of any modular facility to be ACL-2 compliant is another large cost that must be accounted for [35]. Here we infer the cost for an ACL-2 space necessary to rear approximately 1.5 million male culicids per week (Table 2). The cost of each infrastructure item could vary from this estimate depending on the company used, the facility type, previous ownership (versus new), etc.

**Table 2:** Variable names and values used in the R code to derive an estimate of infrastructure and personnel costs

Coded variable	Default variable value
Mosquito and site information	
MaunaLoa.Mos.Sites	Malama Ki; Nanawale; Bryson's; Waiakea; Cooper; Crater; Pu'u
MaunaLoa.Mos.Density.km	4546; 78,547; 14,597; 29,001; 27,615; 1637; 618
MaunaLoa.Elev.m	25; 36; 314; 885; 1024; 1177; 1247
Alakai.Area.ToCntrl.km	117 km^2^
FemalePercent	50% (assumes an equal sex ratio)
Overflooding_Multiplier	10 (10:1 overflooding ratios are commonly used for a control efficacy of 99%; Zheng et al. [11]; Kandul et al. [53])
Basic infrastructure costs	
Year1.Only.Items	ACL2 modification; irradiator; mosquito sex sorters (6); larval rearing units (5); adult cages (100); ovitraps (300); BG traps (50); PCR machine
Year1.Only.Costs	$800,000; $250,000; $6900; $134,500; $11,040; $2400; $7500; $47,000
Electricity	$2000.00 × 12
LaboratorySpace	Median price of an 800 m^2^ modular facility (see Supp. Mat. Section 2 for price quotes)
AllOtherYear.Items	PCR buffers, reagents, primers, Taq, misc. equipment, misc. field supplies
AllOtherYear.Costs	$30,000
Position and personnel costs	
Personnel.Des	Mass rearing; quality control
Wage.Mass.Rearing	$20.00
Wage.Quality.Control	$25.00
HoursPerYear	260 * 8
Fringe	Research Corporation of the University of Hawaiʻi Fringe/Indirect is set at 61.56% for 2018

Another high but potentially optional item (if using only IIT) in the year 1 cost is the irradiator used in IIT/SIT to make the females infertile at such low doses as to not affect male fitness [11]. Sexing costs may vary if the release program is able to use a machine learning/artificial intelligence adult sex selection discriminator, such as that developed by Verily Life Sciences [36]. It is important to note that at this point the cost of the irradiator may be lower than the cost of the Verily technology, but those costs may change. To illustrate the cost differential of the irradiator (Tables 1 and 2) the calculator was run with and without irradiator costs. The highest perpetual costs (year 1 and beyond) are those associated with rearing and quality control personnel (Tables 1 and 2). To rear approximately 1.5 million adult male culicids every week requires eight rearing and three quality control technicians for the extent of the work year (260 days, 8 h/day, Table 2). In the calculator presented here, the default cost per hour of these different positions was higher for the quality control position as it is primarily managerial (Table 2). The wages used here are likely on the low end of the wage spectrum and should be modified as appropriate. All variables used in the calculator can be modified to reflect site-specific information.

### Ratio calculation

In this calculator, the larval rearing units are treated as the primary delimiter defining the production scale of a culicid mass rearing facility. In other words, each incremental increase in the number of larval rearing units necessitates a certain amount of space (for laboratory work, adult rearing, sex sorting, office space, utilities, etc.), positions, and other costs outlined in Table 1. We also assume that each larval rearing unit can rear ~ 1 million culicids/week. Under this assumption, to house one rearing unit or produce ~ 1 million culicids/week (conservatively this equates to ~ 300K IIT males for release/week or a 30% yield) (Table 1), as well as the staff, infrastructure, and space needed to maintain the rearing unit and colony, it is assumed that a facility would need laboratory space of ~ 300–500 m^2^. However, to house five rearing units, it is assumed here that a facility would need a maximum laboratory space of 800 m^2^ [26, 37], thereby incorporating for laboratory size economies of scale. It should be noted that [30] developed their *Cx. quinquefasciatus* rearing methodology for a 70 m^2^ facility, but that facility did not include office or laboratory space. In this calculator, the facility size can be modified to better reflect location-, agency-, or researcher-specific costs.

The rate of increase is calculated and supplied for each projection. This rate can be thought of as both the number of larval rearing units needed as well as the rate of increase for all other items (space, positions, etc.) associated with rearing the necessary number of male culicids (Table 2). The rate is rounded up to the nearest whole unit, from two significant digits of the proportion of wild-type males to laboratory males needed. Conducting the assessment in this way ensures the production capacity needed for the successful implementation of an IIT or IIT/SIT control program. The simplistic but essential Eq. (1) used to define the rate linking production capacity to cost is below:

1 $${r}_{t}=\frac{wmo}{{u}_{t}{n}_{t}}$$

In Eq. (1) the rate of increase at a specific time point (${r}_{t}$) is defined by the interaction of the wild-type culicid population size ($w$), the proportion of males in the wild culicid population targeted ($m$), and the anticipated overflooding ratio ($o$). These ($wmo$) are then divided by the capacity of a single larval rearing unit as defined for a specific time point (${u}_{t}$) multiplied by the proportion of laboratory males reared by that rearing unit at that time (${n}_{t}$). For all projections, estimates were developed using the maximum weekly production ($t$) of IIT males per rearing unit [26, 37].

### Comparison of projection to other studies

We compared the output of this tool in the Alakaʻi Wilderness Reserve to infrastructure costs developed for five IIT/SIT projects from other studies (see Table 3). The studies compared in Table 3 itemized costs that did not include construction or building fees, which would make them equivalent to year 2 and beyond in the calculator reviewed here; therefore, only year 2 projections were compared. These studies also varied widely in the year and country in which the application was conducted. To account for this, all studies reviewed were scaled to the 2022 US dollar (US$) using the average inflation factor estimated from the project year to the 2022 US$ as derived from the Consumer Price Index (CPI) Inflation Calculator developed by the US Bureau of Labor Statistics [38]. All of the studies reviewed in Table 3 incorporate both IIT and SIT production needs into their pipeline, and so are comparable to an IIT/SIT application cost. The cost calculator reviewed here is thought to be site- and species-independent, meaning materials are relatively equivalent for rearing most (if not all) pestiferous culicids, and so cost comparisons were selected based on control application equivalence (IIT/SIT), not targeted species.

**Table 3:** Information from studies used to compare cost estimates of IIT/SIT male production derived elsewhere to that estimated for production in the USA on Kauaʻi

Study	Location	Culicid studied	Year estimated^a,b^	Estimated inflation (to 2022 US$)^c^	Purchasing power parity (to US$)	Published cost (US$)
Singh et al., 1975; 1977	India	*Cx. quinquefasciatus (syn: Cx. fatigans)*	1972–1973	6.95	23.14	$40–50 per million pupae
Zheng et al., 2019	China	*Ae. albopictus*	2016–2017	1.235	4.19	$13–175 to produce 11,640–158,136 HC males/ha/week
Martín-Park et al., 2022	Mexico	*Ae. aegypti*	2019	1.18	10.04	$340 per 4000 IIT/SIT males
Soh et al., 2021	Singapore	*Ae. aegypti*	2021	1.13	0.84	$22.7 million per year across Singapore (2010 US$)
This study	Kauaʻi	*Cx. quinquefasciatus*	2022	1	0.88	–

If multiple years are given for the study, inflation was derived for the average of those years

^a^International purchasing power parity (PPP) derived from the World Bank and accessed through the WDI application in R

^b^Kauaʻi Regional PPP derived from the US Bureau of Economic Analysis: https://www.bea.gov/news/2021/real-personal-consumption-expenditures-and-personal-income-state-2020

^c^Inflation (to 2022 US$) was calculated using the CPI inflation calculator developed by the US Bureau of Labor Statistics: https://www.bls.gov/data/inflation_calculator.html

Though prices were made equivalent to the 2022 US$ when accounting for inflation, the estimated costs still do not account for variations in labor, materials, and other costs between countries and regions. To ensure regional price parity when comparing the infrastructure costs developed for the Alakaʻi Wilderness Reserve to the various studies outlined in Table 3, the 2021 US$ PPP conversion factor developed by the World Bank was obtained for each country using the R application WDI [39]. The 2021 PPP was used as at the time of development it was the latest available. The PPP accounts for price level differences between countries as normalized using the cost of the average US$ [40]. Though the PPP accounts for differences in costs outside of the USA, because it is normalized by the price of the average US$, it does not account for differences in regional US costs. According to the US Bureau of Economic Analysis, Hawaiʻi has the highest regional price parity (RPP) when compared to the average US$ (~ 1.12). Therefore, in Hawaiʻi costs are ~ 12% higher then average [41]. The RPP is used in this analysis in a similar way to the PPP to account for the increased cost of infrastructure development in Hawaiʻi such that cost projections can be more easily compared between Hawaiʻi and other studies.

Throughout this paper we consistently report area in square kilometers due to the use of that metric in the original publication of *Cx. quinquefasciatus* densities [5] in Hawaiʻi. However, when comparing the densities to the costs of other studies, we convert all estimates developed here to hectares (ha), as it was commonly used in the cited costs of IIT/SIT (Table 3).

### Additional examples of utility and reproducibility

In order to maximize the utility of the calculator, and enhance its understanding, two additional examples were completed. The first looks at *Cx. quinquefasciatus* infrastructure needs to develop IIT or IIT/SIT in the home range of ʻiʻiwi *Drepanis coccinea* (Additional file 4: Example report S1), a native Hawaiian honeycreeper federally listed as threatened [42]. The second example shows how, with just a few slight modifications, the calculator can be used to assess the efficacy of IIT or IIT/SIT for control of additional culicids. In this example, the life history characteristics of *Ae. aegypti* are used instead of *Cx. quinquefasciatus* to assess infrastructure needs for IIT or IIT/SIT control on the island of Hawaiʻi (Additional file 5: Example report S2). For both examples, we provide a sample report and all modified R code as run on a Windows 10 computer.

Additionally, we wrote this manuscript and both example reports in R Markdown to maximize reproducibility [43–45] (Additional file 2: Code S2). This methodology is in contrast to other spreadsheet-based cost projections developed for *Aedes *sp*.* SIT, such as those developed by the International Atomic Energy Agency (IAEA) [46, 47]. Though great detail is given in the IAEA tool, a coding-based environment allows for equations and linkages that would make a spreadsheet overly complex. Therefore, for the less refined cost projections estimated here R and R Markdown are more appropriate for this type of application.

## Results and discussion

As implied in Eq. (1) the rate used here may change given certain expectations regarding the life history characteristics of *Cx. quinquefasciatus*. To show this change we compared colonies with an equal sex ratio versus a female-biased sex ratio (Tables 4 and 5, respectively), and the cost of control does not change (first-year Puʻu costs; Table 4 vs. Table 5): $1,162,937.00 vs. $1,162,937.00). In fact, the change between sex ratios is relatively similar for the analysis no matter the starting density (Table 4 vs. Table 5). The comparison between these assessments shows that this calculator is not necessarily sensitive to fluctuations in sex ratio due to the cost buffering associated with linking costs to the number of larval rearing units. In this analysis, the conservative estimate of male yield per larval rearing unit (30%) buffers the variation in sex ratio, thus reducing its effect on rearing costs. Notably, colonies used for colony maintenance or expansion may be artificially skewed toward a female-biased sex ratio to decrease male harassment and increase female egg laying [48].

**Table 4:** Calculator output for male southern house mosquito *Cx. quinquefasciatus* IIT/SIT mass release production needs as projected for a 117 km^2^ area

Site	Elevation (m)^a^	Wild-type males	IIT:Wild-type males (10:1)^b^	Rate used	First-year cost ($)^c^	Subsequent year costs ($)
Malama Ki	25	265,941.0	2,659,410	9	$4,240,716.00	$1,691,470.00
Nanawale	36	4,594,999.5	45,949,995	150	$66,605,273.00	$28,191,158.00
Bryson's	314	853,924.5	8,539,245	28	$12,655,118.00	$5,262,350.00
Waiakea	885	1,696,558.5	16,965,585	57	$25,493,204.00	$10,712,640.00
Cooper	1024	1,615,477.5	16,154,775	54	$24,147,298.00	$10,148,817.00
*Crater*	*1177*	*95,764.5*	*957,645*	*4*	*$2,028,874.00*	*$751,764.00*
*Pu'u*	*1247*	*36,153.0*	*361,530*	*2*	*$1,162,937.00*	*$375,882.00*

Each row represents a proxy location in which densities have been defined. The elevation of the proxy site is shown, as well as the number of wild-type males projected into an equivalent site with an area of 117 km^2^. Italicized areas have elevations equivalent to the Alakaʻi Wilderness Reserve. An overflooding ratio of 10:1 is used to infer the amount of IIT mosquitoes needed to control mosquito densities at the proxy site. The “rate used” column identifies how many larval rearing units (and their associated infrastructure) are necessary to rear the number of IIT males. In the table, a rate of 1 is equivalent to the production of ≤ 1 million IIT/SIT culicids using the default (1:1) sex ratio

^a^Kokee State Park Visitors Center elevation is ~ 1115 m; Alakaʻi Swamp elevation is ~ 1219–1402 m

^b^Alakaʻi Wilderness Reserve with a 2 km buffer has a combined area of 117 km^2^

^c^This does not include mosquito dispersal/application costs

**Table 5:** The male southern house mosquito *Cx. quinquefasciatus* IIT/SIT mass release production needs for a 117 km^2^ area

Site	Elevation (m)^a^	Wild-type males	IIT:Wild-type males (10:1)^b^	Rate used	First year-cost (US$)^c^	Subsequent year costs (US$)
0.43:1 male/female sex ratio						
Nanawale	36	4,594,999.5	45,949,995	150	$66,605,273.00	$28,191,158.00
*Crater*	*1177*	*95,764.5*	*957,645*	*4*	*$2,028,874.00*	*$751,764.00*
*Pu'u*	*1247*	*36,153.0*	*361,530*	*2*	*$1,162,937.00*	*$375,882.00*
No laboratory costs added						
Nanawale	36	4,594,999.5	45,949,995	150	$55,841,358.00	$28,191,158.00
*Crater*	*1177*	*95,764.5*	*957,645*	*4*	*$1,741,836.00*	*$751,764.00*
*Pu'u*	*1247*	*36,153.0*	*361,530*	*2*	*$1,019,418.00*	*$375,882.00*
No irradiator costs added						
Nanawale	36	4,594,999.5	45,949,995	150	$66,355,273.00	$28,191,158.00
*Crater*	*1177*	*95,764.5*	*957,645*	*4*	*$1,778,874.00*	*$751,764.00*
*Pu'u*	*1247*	*36,153.0*	*361,530*	*2*	*$912,937.00*	*$375,882.00*

Each row represents a proxy location in which densities have been defined. The elevation of the proxy site as well as the number of wild-type males projected into an equivalent site with an area of 117 km^2^ is shown. Italicized areas have elevations equivalent to the Alakaʻi Wilderness Reserve. An overflooding ratio of 10:1 is used to infer the amount of IIT mosquitoes needed to control mosquito densities at the proxy site. The “rate used” column identifies how many larval rearing units (and their associated infrastructure) are necessary to rear the number of IIT males. In the table a rate of 1 is equivalent to the production of ≤ 1 million IIT/SIT culicids using the default (1:1) sex ratio

^a^Kokee State Park Visitors Center elevation is ~ 1115 m; Alakaʻi Swamp elevation is ~ 1219–1402 m

^b^Alakaʻi Wilderness Reserve with a 2 km buffer has a combined area of 117 km^2^

^c^This does not include mosquito dispersal/application costs

As noted in the methods section, two other costs that require closer examination are the costs of the mobile laboratory and the irradiator (Table 2). Both the mobile laboratory and irradiator are crucial to an IIT/SIT effort [11], but matching efforts from other institutions or organizations could offset, and thus reduce, projected costs. Table 5 shows the infrastructure costs for a subset of sites for the IIT/SIT effort without the cost of a rearing facility, which for the Alakaʻi Wilderness Reserve is at least $1,741,836.00, with subsequent yearly costs of $751,764.00. Note how the subsequent yearly costs stay constant; this is because the laboratory costs are assessed in the first year. As implied earlier, there are also potential cost offsets associated with using a possibly more efficient and effective sex sorting mechanism without the use of radiation [36]. Table 5 also shows the cost of all other default infrastructure costs without the irradiator (i.e., just IIT), which for the Alakaʻi Wilderness Reserve is at least $912,937.00, with subsequent yearly costs of $375,882.00. Something that should be noted when removing both rearing facility and irradiator costs (Table 5) is that the rate of infrastructure increase is the same (rate of increase at Puʻu: 2). This rate invariance is true for all assessments, even those that deal with alternative life history characteristics, such as differences in sex ratios. This is because the rate acts as a biologically informed proportion from which these unit costs are estimated and is buffered by the conservative estimate of male yield (30%).

When comparing year 2 costs derived from other studies to those estimated in Hawaiʻi using the targeted treatment amounts (average cost/ha/year), the estimates derived here were lower due to lower projected treatment needs (Table 6). However, when the cost per 1000 IIT males was defined per study, and inflation and PPP were accounted for, the costs estimated here are on the higher end (Table 6). Throughout this analysis we have used conservative cost estimates, and have not attempted to reduce those costs when comparing them to additional studies. Costs can (and should) be optimized by users of the calculator with better understanding of the location-specific costs. For instance, if fringe benefits were reduced to ~ 17.5%, an amount associated with conservation-based Cooperative Ecosystem Study Units [49], the average cost per 1000 IIT/SIT males produced in Hawaiʻi falls to $15.42. However, for this initial comparison, production costs on the higher end of the spectrum are to be expected and applied until the location-specific costs can be optimized. Though this is not a statistical comparison, and inflation and PPP estimates have their own assumptions [38, 40], this does give some support to the relevance of the cost projection in assessing and comparing the cost of IIT/SIT. Also, as noted in “Additional examples of utility and reproducibility,” the IAEA SIT cost spreadsheets [46, 47] are essential documents that should be reviewed when itemizing a detailed cost plan. In comparison, many of the recommendations in the IAEA spreadsheet and subsequent methodological write-up are applicable to culicid control using IIT and IIT/SIT. Though these two cost projection estimators (IAEA versus that developed here) differ in their connectivity between elements and specificity, they are both structures that can be improved upon and updated as location-specific information become available. We suggest here that the methodological and reproducibility framework that R offers may benefit researchers interested in understanding and modifying the cost estimator. The R environment allows easier iterative replications, connectivity of specific elements and linking of those elements to the area of control. Though the tool produced here attempts to link targeted landscapes to control cost, this is only the first step at doing so. If available, more geographically explicit variables could be run through a modified version of the cost calculator presented here for a more robust projection of costs.

**Table 6:** Comparison of infrastructure costs developed for other Culicidae with that developed for *Cx. quinquefasciatus* here

Study	Average cost/ha/year (2022 US$)	Average cost per 1000 IIT/SIT males (2022 US$)	Cost equivalence to Kauaʻi for 1000 IIT/SIT males using PPP (2022 US$)
Singh et al. 1975; 1977	*–*	$0.37	$7.60
Zheng et al. 2019	*$5,767.84*	$1.31	$4.84
Martín-Park et al. 2022	*$436.70*	$2.10	$18.56
Soh et al. 2021^1^	*$361.80*	–	–
This study	*$32.13*	$20.00	$20.00

The cost per hectare (ha) per year, and the cost per 1000 IIT/SIT males produced is used as a basis for comparing previous estimates to that projected for Kauaʻi. The PPP is the purchasing power of the average US dollar in relation to a country or state. Here the PPP is used to control for the variation in international and state purchasing power, in relation to the Kauaʻi projection. The values used for the Kauaʻi projection are the averages of the Crater and Puʻu sites, areas that represent primary forest bird refugia

^1^Total Singapore treatment area (7900 ha) for Soh et al. (2022) was derived from the World Bank table found here: https://data.worldbank.org/indicator/AG.LND.TOTL.K2?locations=SG

There are some caveats to this assessment that when applied could potentially modify the projected costs associated with the application of IIT, or IIT/SIT. First, this analysis only estimates the cost of culicid-rearing infrastructure and the personnel needed for that infrastructure. The analysis does not consider costs associated with field release and monitoring, or community outreach; both will significantly increase the cost of application. Using infrastructure developed elsewhere, and shipping the viable males as needed, can decrease infrastructure costs and time to implementation, but the costs of outsourcing may be greater than development of a more localized facility. The costs of outsourcing such a facility are both tangible (e.g., greater cost per unit, direct local investment, decreased tool efficacy due to long transit times) and intangible (e.g., community involvement and support, the development of scientific leadership and knowledge specific to the community, ecology, and organism(s) of interest), and should be accounted for. However, in the time it takes to develop a rearing facility and expertise required to mass rear *Cx. quinquefasciatus* for a conservation application, extinctions may occur. Therefore, a hybrid approach may be best suited to expedite the tools application. Whether outsourced or not, the benefit of developing and enhancing *Cx. quinquefasciatus* landscape-scale control in Hawaiʻi using IIT or IIT/SIT is considerable.

Another important aspect to be mindful of is that captive rearing and landscape-scale control applications are not mutually exclusive. Until the infrastructure, outreach, and release capabilities of IIT or IIT/SIT are optimized over all forest bird refugia, captive rearing will be a necessary backstop preventing extinction. Therefore, the infrastructure costs needed to develop IIT or IIT/SIT cannot (at least initially) offset or re-purpose the costs of captive rearing. In Hawaiʻi, captive rearing and landscape-scale mosquito control tools should be used to the maximum extent practicable to ensure the survival of the resources they are designed to protect.

Both IIT and SIT have been used to successfully suppress, eradicate, contain, and prevent establishment of insects of health and agricultural concern [8, 11–16, 50]. From the nearly successful eradication of *Cx. quinquefasciatus* [10] using IIT, to the canonical use of SIT to eliminate screwworms [50, 51], these tools have proven to be powerful additions to any IPM plan [52]. Although the costs of implementing an IIT or SIT tool in perpetuity may seem great, generally the benefit that the tool provides outweighs those costs [21, 52]. For instance, the annual economic benefit to the United States from screwworm eradication using SIT is ~ $2.8 billion annually whereas the program ultimately cost $32 million as of 2016 [51]. However, the economic benefit of the screwworm eradication program is relatively easy to calculate compared to the conservation application of IIT or IIT/SIT in Hawaiʻi. For Hawaiʻi’s native forest birds, the cost of inaction is extinction.

## Conclusions

Here we show the utility of a cost estimate calculator for both IIT and IIT/SIT mass rearing infrastructure adapted for Hawaiʻi from [26] and [11]. The calculator uses a simplified linear relationship of rearing infrastructure to cost; this pragmatic approach allows for the development of initial culicid mass rearing infrastructure cost estimates. The benefit of using this approach is that it links all infrastructure needs into a single cost calculator and allows interested non-specialists to simulate regional infrastructure costs. As a proof of concept, we estimated *Cx. quinquefasciatus*-rearing infrastructure costs using the IIT or the IIT/SIT approach [11] as applied to an area the size of the Alakaʻi Wilderness Reserve. To control *Cx. quinquefasciatus* in and around the Alakaʻi Wilderness Reserve, minimum first-year costs were estimated to be approximately $1.16M with subsequent yearly costs of approximately $376K. The estimates of infrastructure cost presented here are a critical step in the development of an effective IPM implementation plan.

## Supplementary Information

**Additional file 1: Code S1.** Code used to infer infrastructure costs. The file contains two versions of the same code. The first outputs an .html document that will be placed in a folder called *MosquitoCosts* on the desktop of your computer. The *MosquitoCosts* folder will be automatically generated by the code. The .html document produced by this code should open automatically as a tab on your internet browser. The second version is the same code in the form of a function. The function outputs a table to an integrated development environment (IDE) such as RStudio, and can be used in R to enable additional modifications and report development.

**Additional file 2: Code S2.** A folder containing all of the R markdown code used to develop the manuscript and example reports. The contents of this folder can be used to reproduce all analyses and reports presented here.

**Additional file 3: Quotes S1. **Quotes derived from the Federal GSA advantage website.

**Additional file 4: Example report S1.** Release cost estimate for control of *Cx. quinquefasciatus* within ʻiʻiwi’s home range.

**Additional file 5: Example Report S2.** Release cost estimate for control of *Ae. aegypti* on the island of Hawaiʻi.

## Data Availability

All data and code used to derive these projections are freely available within the supplementary materials.
