# Supplementary material for: A mass rearing cost calculator for the control of Culex quinquefasciatus in Hawaiʻi using the incompatible insect technique
Source: Parasit Vectors. 2022 Dec 5;15:453. doi: 10.1186/s13071-022-05522-1 (PMC9724328; doi:10.1186/s13071-022-05522-1)
Supplement: Supplementary file 1 — Additional file 1: Code S1. Code used to infer infrastructure costs. The file contains two versions of the same code. The first outputs an .html document that will be placed in a folder called MosquitoCosts on the desktop of your computer. The MosquitoCosts folder will be automatically generated by the code. The .html document produced by this code should open automatically as a tab on your internet browser. The second version is the same code in the form of a function. The function outputs a table to an integrated development environment (IDE) such as RStudio, and can be used in R to enable additional modifications and report development. [file 13071_2022_5522_MOESM1_ESM.zip › 1-Code_S1_PandV3.docx]

A mass-rearing cost calculator for the control of *Culex quinquefasciatus* in Hawaiʻi using IIT

**Adam E. Vorsino^1^***, **Zhiyong Xi^2^**

^1^Strategic Habitat Conservation Program, Ecological Services, Pacific Islands Fish and Wildlife Office, U.S. Fish and Wildlife Service, 300 Ala Moana Blvd Ste. 3-122, Honolulu, Hawaiʻi 96850

^2^Department of Microbiology and Molecular Genetics, Michigan State University, 314 Giltner Hall, 293 Farm Lane, East Lansing, Michigan 48824

*Corresponding author: [Adam_Vorsino@fws.gov](mailto:Adam_Vorsino@fws.gov)

AEV: [Adam_Vorsino@fws.gov](mailto:Adam_Vorsino@fws.gov)

ZX: [xizy@msu.edu](mailto:xizy@msu.edu)

# Supplemental Materials Section 1: Code used to infer infrastructure costs

Below are two versions of the same code. The first is the function used throughout this manuscript to assess costs and compare sites. It can be made to output a table to an Integrated Development Environment (IDE) such as [Rstudio], and can be used in R to enable additional modifications and report development. The second code outputs an *.html* document that will be placed in a folder called *MosquitoCosts* on the desktop of your computer. The *MosquitoCosts* folder will be automatically generated by the code. The html document produced by this code should open automatically as a tab on your internet browser.

## Cost estimator function with table output for *C. quinquefasciatus* cost calculator

CostEst <- function(FemalePercent, # Percent used to determine sex ration. Percent of the laboratory reared individuals that are female.
 Overflooding_Multiplier, # The number of IIT males to be released in relation to a single wild-type
 Area, # The size of the area to treat
 LaboratorySpace, # predicted cost of an area necessary to rear 1.5 million male IIT
 Irradiator, # The Cost of an irradiator
 FringeCst = NULL, # Technicians and workers Indirect costs/fringe: NULL = 0.6156
 Wage.Mass.Rearing = NULL, # Mass rearing worker wages (per hour): NULL = 20
 Wage.Quality.Control = NULL, #QC workers wages (per hour): NULL = 25
 HoursPerYear = NULL, # Hours that colonies must be maintained per year (to calculate yearly wages): NULL = 260 * 8
 ACL2 = NULL,# Cost of upgrading a standard rearing area necessary to rear 1.5 million IIT males (i.e. LaboratorySpace) to an ACL-2 facility NULL = 800000
 Electricity, # estimated yearly electricity usage to rear 1.5 million IIT males/year
 Table = F) # run to export table (T or F)
 {


 if(is.null(ACL2)){
 ACL2 <- 800000
 }

 LaboratorySpace <- LaboratorySpace + ACL2

 # Ecology and Biology
 MaunaLoa.Mos.Density.km <- c(4546,78547,14597,29001,27615,1637,618)#,0,0)
 MaunaLoa.Mos.Sites <- c('Malama Ki','Nanawale',"Bryson's",'Waiakea','Cooper','Crater',"Pu'u")#,'CJR',"Solomon's")
 MaunaLoa.Elev.m <- c(25, 36, 314, 885, 1024, 1177, 1247)#, 1678, 1686)
 Alakai.Area.ToCntrl.km <- Area# kilometers squared
 # FemalePercent <- 70 # Sex ratio
 # Overflooding_Multiplier <- 10 # Multiplier to derive the ratio needed for control

 # Operations/Supplies Costs
 Year1.Only.Items <- c('Irradiator', 'Mosquito.Sex.Sorters (6)', 'larvae Rearing Units (5)',
 'adult cages (100)', 'ovitraps (300)', 'BG traps (50)', 'PCR Machine', 'ACL-2 upgrade')#, 'modulare office (60x24ft)')
 Year1.Only.Costs <- c(Irradiator, 6900, 134500, 11040, 2400, 7500, 47000)#, 40000)

 # Personel Costs
 Personel.Des <- c('Mass Rearing', 'Quality Control')#, 'Feild Release', 'Community Education')
 if(is.null(Wage.Mass.Rearing)){
 Wage.Mass.Rearing <- 20 #15 dollars/hour
 }
 if(is.null(Wage.Quality.Control)){
 Wage.Quality.Control <- 25 #17 dollars/hour
 }

 if(is.null(HoursPerYear)){
 HoursPerYear <- 260 * 8 # 260 is 52 weeks/year 5 days/week * 8 hours per day
 }

 if(is.null (FringeCst)){
 Fringe <- 0.6156
 }else{
 Fringe <- FringeCst
 }

 # Misc Costs
 AllOtherYear.Items <- 'Misc PCR/Lab/Field Supplies'
 AllOtherYear.Costs <- 30000 # ensureing that it only changes for every 0.5 increment

 ##################################################################################
 ###########DO NOT MODIFY PAST THIS SECTION########################################
 ##################################################################################

 pckgs <- c('stringr', 'kableExtra', 'Hmisc', 'formattable', 'flextable')

 for(pck in pckgs){
 if ((is.na(installed.packages()[,"Package"][pck]))==T){
 install.packages(pck, dependencies = T)
 }
 library(pck,character.only = T)

 }

 #Bio
 r = 1-(FemalePercent/100) #male proportion given female percent
 wildtypefmales <- 0.5
 WildType.Males.km <- (((Alakai.Area.ToCntrl.km*MaunaLoa.Mos.Density.km)) * wildtypefmales)
 Wolb.Males.Needed.km <- round(WildType.Males.km*Overflooding_Multiplier)

 #Office
 OfficeCosts <- LaboratorySpace + Electricity

 #Personel
 Wage <- c(Wage.Mass.Rearing, Wage.Quality.Control)
 Personel.CossA <- rep(HoursPerYear*Wage)
 Personel.Costs <- Personel.CossA
 Fringe.Costs <- Personel.Costs * Fringe
 Tot.Pers.Costs <- (Personel.Costs + Fringe.Costs) * c(8,3)


 #COSTS FOR A Facility 1-300k males
 Year1.Only.Costs.300k <- Year1.Only.Costs
 Year1.Only.Costs.300k[2:6] <- Year1.Only.Costs[2:6]/5
 OfficeCosts.300k <- OfficeCosts/5
 Tot.Pers.Costs.300k <- (Personel.Costs + Fringe.Costs) * (c(8,3)/5)
 AllOtherYear.Costs.300k <- AllOtherYear.Costs/5

 MaxCeiling <- 1000000 * (0.3)#(r)# 1 million Culicid per rearing unit, 30% male production

 Wolb.Males.Produced <- (Wolb.Males.Needed.km/wildtypefmales) * r
 PropWildToLab <- Wolb.Males.Produced/Wolb.Males.Needed.km
 diff.ratio <- ceiling(signif(round(Wolb.Males.Needed.km/MaxCeiling, 2),2))


 # ensureing a min of a million mailes to be reared
 # diff.ratio[which(diff.ratio < round(500000/MaxCeiling))] <- ceiling(500000/MaxCeiling)

 # Defining the numbers for each scenario
 AmountNeeded <- data.frame()
 for(rate in diff.ratio){

 # YEAR 1 COSTS
 Yr1.ItemCostJnk <- Year1.Only.Costs.300k
 Yr1.ItemCostJnk[2:6] <- Year1.Only.Costs.300k[2:6] * rate
 rate2 <- ceiling(rate/5)
 Yr1.ItemCostJnk[7] <- Year1.Only.Costs.300k[7] * rate2

 Yr1.PersCostJnk <- (Personel.Costs + Fringe.Costs) * ((c(8,3)/5)*rate)
 Yr1.MiscJnk <- AllOtherYear.Costs.300k*rate

 rate3 <- rate
 Yr1.OfficeCostsJnk <- OfficeCosts.300k * rate3
 Yr1.TotalCosts <- currency(sum(Yr1.ItemCostJnk, Yr1.PersCostJnk, Yr1.MiscJnk, Yr1.OfficeCostsJnk))


 # SUBSEQUENT YEAR COSTS
 SUB.PersCostJnk <- (Personel.Costs + Fringe.Costs) * ((c(8,3)/5)*rate)
 SUB.MiscJnk <- AllOtherYear.Costs.300k*rate
 SUB.OfficeCostsJnk <- Electricity * rate3
 SUB.TotalCosts <- currency(sum(SUB.PersCostJnk, SUB.MiscJnk, SUB.OfficeCostsJnk))

 AmountNeeded <- rbind(AmountNeeded, cbind(rate, round(Yr1.TotalCosts), round(SUB.TotalCosts)))

 }
 AmountNeeded$V3 <- currency(AmountNeeded$V3)
 AmountNeeded$V2 <- currency(AmountNeeded$V2)


 AmountNeeded2 <- data.frame(cbind(MaunaLoa.Mos.Sites, MaunaLoa.Elev.m, WildType.Males.km, Wolb.Males.Needed.km, AmountNeeded))#

 colnames(AmountNeeded2) <- capitalize(c('Equivalent Site', 'Elevation (m)', 'Wild Type Males', 'IIT:Wild Type Males (10:1)',
 'Rate Used', 'First Year Cost ($)', 'Subsequent Year Costs ($)'))


 Partitions <- c(5,7)
 AmountNeeded3 <- AmountNeeded2
 if(Table == T){
 PrCntrlTable1 <- flextable(data.frame(AmountNeeded3)) %>%
 italic(i = c(6:7), italic = TRUE, part = "body") %>%
 set_header_labels(Equivalent.Site = "Site",
 Elevation..m. = 'Elevation (m)',
 Wild.Type.Males = 'Wild Type Males',
 IIT.Wild.Type.Males..10.1. = 'IIT:Wild Type Males (10:1)',
 Rate.Used = 'Rate Used',
 First.Year.Cost.... = 'First Year Cost ($)',
 Subsequent.Year.Costs.... = 'Subsequent Year Costs ($)') %>%

 theme_booktabs(bold_header = T) %>%
 flextable::align(align = "center", part = "header") %>%
 valign(valign = "center", part = "all") %>%
 hline(i=c(7), part = "body") %>%
 hline(i=c(1), part = "header", border = officer::fp_border()) %>%
 hline_top(part="header", border = officer::fp_border()) %>%
 fontsize(part = "header", size = 12) %>%
 flextable::align(align = 'center', part = 'all') %>%
 colformat_int(j = 2:7, big.mark = ",") %>%
 autofit() %>%
 fontsize(size = 12, part = 'body') %>%
 fit_to_width(max_width = 7.25) %>%

 footnote(value = as_paragraph(c('Kokee State Park Visitors Center Elevation is ~1,115 meters; Alakai Swamp Elevation is ~1,219-1,402 meters.',
 paste0('Alakai Wilderness Reserve with a 2 kilometer buffer has a combined area of ', round(TreatementArea), ' kilometers squared.'),
 'This does not include mosquito dispersal/application costs.')), part = 'header', i = 1, j = c(2,4,6),
 ref_symbols = c('1', '2', '3')) %>%
 fontsize(part = 'footer', size = 8) %>%
 italic(part = 'footer', italic = T)#,

 PrCntrlTable1
 }else{
 AmountNeeded3
 }
}


# Example used for Table 1 in manuscript

# LaboratorySpaceA <- 800 * median(c((171000/((72*60)* 0.092903)),
# (150000/((60*60)* 0.092903)),
# (180000/((60*48)* 0.092903))))
#
# IrraditatorA <- 250000
#
# see1 <- CostEst(FemalePercent = 50,
# Overflooding_Multiplier = 10,
# Area = round(TreatementArea),
# LaboratorySpace = LaboratorySpaceA,
# Irradiator = IrraditatorA,
# Electricity = 2000 * 12,
# Table = T)
#
# see1

## Cost estimator code with HTML table output for *C. quinquefasciatus* cost calculator

rm(list = ls())
try(graphics.off(), T)

# Ecology and Biology
MaunaLoa.Mos.Density.km <- c(4546,78547,14597,29001,27615,1637,618)
MaunaLoa.Mos.Sites <- c('Malama Ki','Nanawale',"Bryson's",'Waiakea','Cooper','Crater',"Pu'u")
MaunaLoa.Elev.m <- c(25, 36, 314, 885, 1024, 1177, 1247)
Alakai.Area.ToCntrl.km <- 24
FemalePercent <- 50
Overflooding_Multiplier <- 10
# Operations/Supplies/Office Costs
Year1.Only.Items <- c('Irradiator', 'Mosquito.Sex.Sorters (6)', 'larvae Rearing Units (5)',
 'adult cages (100)', 'ovitraps (300)', 'BG traps (50)', 'PCR Machine')#, 'modulare office (60x24ft)')
Year1.Only.Costs <- c(200000, 6900, 134500, 11040, 2400, 7500, 47000)#, 40000)

Electricity <- 2000 * 12

ACL2 <- 800000

LaboratorySpace <- 800 * median(c((171000/((72*60)* 0.092903)),
 (150000/((60*60)* 0.092903)),
 (180000/((60*48)* 0.092903))))

# Personel Costs
Personel.Des <- c('Mass Rearing', 'Quality Control')
Wage.Mass.Rearing <- 15
Wage.Quality.Control <- 17
HoursPerYear <- 260 * 8

Fringe <- 0.6156

# Misc Costs
AllOtherYear.Items <- 'Misc PCR/Lab/Field Supplies'
AllOtherYear.Costs <- 30000


########################################################################
#DO NOT MODIFY PAST THIS SECTION########################################
########################################################################

pckgs <- c('stringr', 'kableExtra', 'Hmisc', 'formattable', 'flextable')

for(pck in pckgs){
 if ((is.na(installed.packages()[,"Package"][pck]))==T){
 install.packages(pck, dependencies = T)
 }
 library(pck,character.only = T)

}

WorkFolder <- paste0(file.path(Sys.getenv("USERPROFILE"),"Desktop"), '/MosquitoCosts/') # Desktop location
if(dir.exists(WorkFolder) == F){
 dir.create(WorkFolder)
}


setwd(WorkFolder)

#Bio
r = 1-(FemalePercent/100) #male proportion given female percent
wildtypefmales <- 0.5
WildType.Males.km <- (((Alakai.Area.ToCntrl.km*MaunaLoa.Mos.Density.km)) * (wildtypefmales)) #(Samuels et al. 2014 numbers)
Wolb.Males.Needed.km <- round(WildType.Males.km*Overflooding_Multiplier)

#Office
OfficeCosts <- LaboratorySpace + Electricity + ACL2

#Personel
Wage <- c(Wage.Mass.Rearing, Wage.Quality.Control)
Personel.CossA <- rep(HoursPerYear*Wage)#, length(Personel.Des)) # 260 hours per year
Personel.Costs <- Personel.CossA # removing 6 positions because community education and feild release
Fringe.Costs <- Personel.Costs * Fringe
Tot.Pers.Costs <- (Personel.Costs + Fringe.Costs) * c(8,3)


#COSTS FOR A Facility 1-300k males
# (defined by assuming that each 1 mosquito larvae rearing unit can
# make 300k males (1 million total)
Year1.Only.Costs.300k <- Year1.Only.Costs
Year1.Only.Costs.300k[2:6] <- Year1.Only.Costs[2:6]/5
OfficeCosts.300k <- OfficeCosts/5 # 800 m^2 facility/2 = 400m^2 facility
Tot.Pers.Costs.300k <- (Personel.Costs + Fringe.Costs) * (c(8,3)/5)
AllOtherYear.Costs.300k <- AllOtherYear.Costs/5

MaxCeiling <- 1000000 * (0.3)# 1 million Culicid per rearing unit, 30% male production

Wolb.Males.Produced <- (Wolb.Males.Needed.km/wildtypefmales) * r # divide by wildtypefmales to put it back into male/female amount

PropWildToLab <- Wolb.Males.Produced/Wolb.Males.Needed.km
diff.ratio <- ceiling(signif(round(Wolb.Males.Needed.km/MaxCeiling, 2),2))

# Defining the numbers for each scenario
AmountNeeded <- data.frame()
for(rate in diff.ratio){

 # YEAR 1 COSTS
 Yr1.ItemCostJnk <- Year1.Only.Costs.300k
 Yr1.ItemCostJnk[2:6] <- Year1.Only.Costs.300k[2:6] * rate
 # PCR machine is for each 1.5 million so
 rate2 <- ceiling(rate/5)
 Yr1.ItemCostJnk[7] <- Year1.Only.Costs.300k[7] * rate2

 Yr1.PersCostJnk <- (Personel.Costs + Fringe.Costs) * ((c(8,3)/5)*rate)
 Yr1.MiscJnk <- AllOtherYear.Costs.300k*rate


 rate3 <- rate#ceiling(rate/2.5)
 Yr1.OfficeCostsJnk <- OfficeCosts.300k * rate3
 Yr1.TotalCosts <- currency(sum(Yr1.ItemCostJnk, Yr1.PersCostJnk, Yr1.MiscJnk, Yr1.OfficeCostsJnk))


 # SUBSEQUENT YEAR COSTS
 SUB.PersCostJnk <- (Personel.Costs + Fringe.Costs) * ((c(8,3)/5)*rate)
 SUB.MiscJnk <- AllOtherYear.Costs.300k*rate
 SUB.OfficeCostsJnk <- Electricity * rate3
 SUB.TotalCosts <- currency(sum(SUB.PersCostJnk, SUB.MiscJnk, SUB.OfficeCostsJnk))

 AmountNeeded <- rbind(AmountNeeded, cbind(rate, round(Yr1.TotalCosts), round(SUB.TotalCosts)))#Alakai.Area.ToCntrl.km,

}
AmountNeeded$V3 <- currency(AmountNeeded$V3)
AmountNeeded$V2 <- currency(AmountNeeded$V2)


AmountNeeded2 <- data.frame(cbind(MaunaLoa.Mos.Sites, MaunaLoa.Elev.m, WildType.Males.km, Wolb.Males.Needed.km, AmountNeeded))#

colnames(AmountNeeded2) <- capitalize(c('Equivalent Site', 'Elevation (m)', 'Wild Type Males', 'IIT:Wild Type Males (10:1)',
 'Rate Used', 'First Year Cost ($)', 'Subsequent Year Costs ($)'))
for(tocomma in 2:4){
 AmountNeeded2[,tocomma] <- comma(AmountNeeded2[, tocomma], digits = 0L)
}


Partitions <- c(5,7)
#'Area of Control (sqr. km.)',
PrCntrlTable <- kable(AmountNeeded2,
 caption = paste0('<b>IIT/SIT Male <i>Culex quinquefasciatus</i> Mass Release Production and Costs for a ',
 Alakai.Area.ToCntrl.km,
 ' km<sup>2</sup> area. In the table a rate of 1 is equivalent to the production of &le; 500,000 IIT/SIT males.
 This does NOT include costs associated with community outreach or field releases.</b>'),
 escape = F, format = 'html') %>%
 kable_styling("striped", full_width = F) %>%
 group_rows(toupper(paste0("Low Elevation")), 1, Partitions[1]-1, indent = F) %>%
 group_rows(toupper(paste0("Mid Elevation")), Partitions[1], Partitions[2], indent = F) %>%
 row_spec(row = 0, bold = T, align = 'center') %>%
 row_spec(row = 1:nrow(AmountNeeded2), align = 'center') %>%
 add_footnote(c('Note: Kokee State Park Visitors Center Elevation is ~1,115 meters; Alakai Swamp Elevation is ~1,219-1,402 meters.',
 'Note: Kokee State Park and Kuia Natural Area Reserve have a combined area of 24 square kilometers.',
 'Note: This does not include mosquito dispersal/application costs.'), notation = 'number')

kableExtra:::as_image(PrCntrlTable, file = paste0(WorkFolder, 'Rearing Costs for ',
 Alakai.Area.ToCntrl.km , 'sqr.km_1.html'))
