# Supplementary material for: A mass rearing cost calculator for the control of Culex quinquefasciatus in Hawaiʻi using the incompatible insect technique
Source: Parasit Vectors. 2022 Dec 5;15:453. doi: 10.1186/s13071-022-05522-1 (PMC9724328; doi:10.1186/s13071-022-05522-1)
Supplement: Supplementary file 3 — Additional file 3: Quotes S1. Quotes derived from the Federal GSA advantage website. [file 13071_2022_5522_MOESM3_ESM.docx]

A mass-rearing cost calculator for the control of *Culex quinquefasciatus* in Hawaiʻi using IIT

**Adam E. Vorsino^1^***, **Zhiyong Xi^2^**

^1^Strategic Habitat Conservation Program, Ecological Services, Pacific Islands Fish and Wildlife Office, U.S. Fish and Wildlife Service, 300 Ala Moana Blvd Ste. 3-122, Honolulu, Hawaiʻi 96850

^2^Department of Microbiology and Molecular Genetics, Michigan State University, 314 Giltner Hall, 293 Farm Lane, East Lansing, Michigan 48824

*Corresponding author: [Adam_Vorsino@fws.gov](mailto:Adam_Vorsino@fws.gov)

AEV: [Adam_Vorsino@fws.gov](mailto:Adam_Vorsino@fws.gov)

ZX: [xizy@msu.edu](mailto:xizy@msu.edu)

# *Supplemental Materials Section 2:* Quotes used to derive facility per meter cost estimate

The following quotes were derived from the [Federal GSA advantage website](https://www.gsaadvantage.gov/advantage/main/start_page.do). Each of the three quotes were converted from square foot to meters squared, and the median of all three was used to estimate the cost of an 800m^2^ modular facility. An 800m^2^ facility is assumed to be large enough for rearing approx. 1.5 million IIT/SIT male Culicid. It has been recommended that *at minimum* the facility size should be optimized to rear at least 500k - 1 million IIT/SIT male Culicid (Dr. Zhiyong Xi, *pers. comm*) as cost savings are insignificant (and may be detrimental to the process) below that level. The maximum recommended size of a facility necessary to rear at least 500k - 1 million IIT/SIT male Culicid is 300-500m^2^ (Dr. Zhiyong Xi, *pers. comm*).


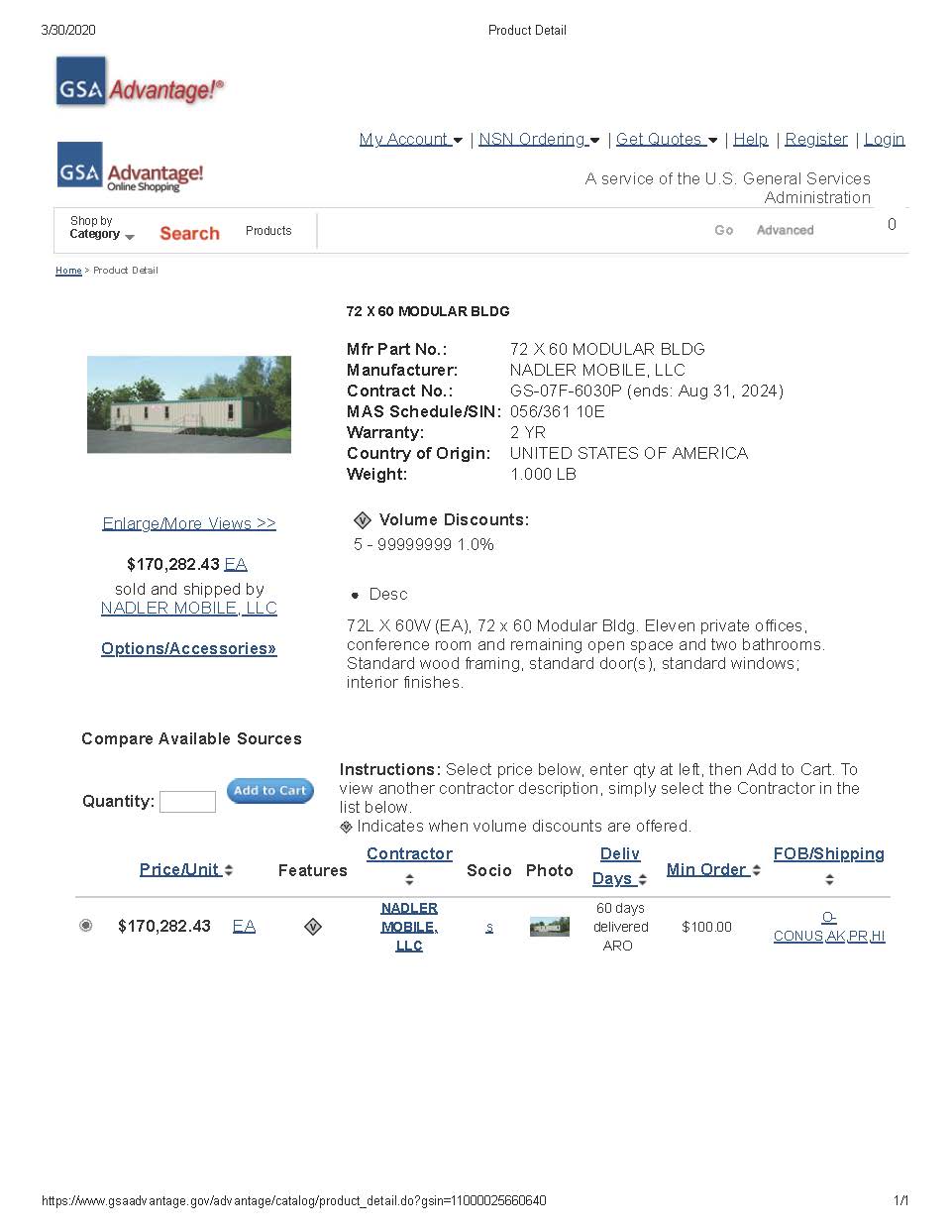


Figure 1: Modular office quote 1.


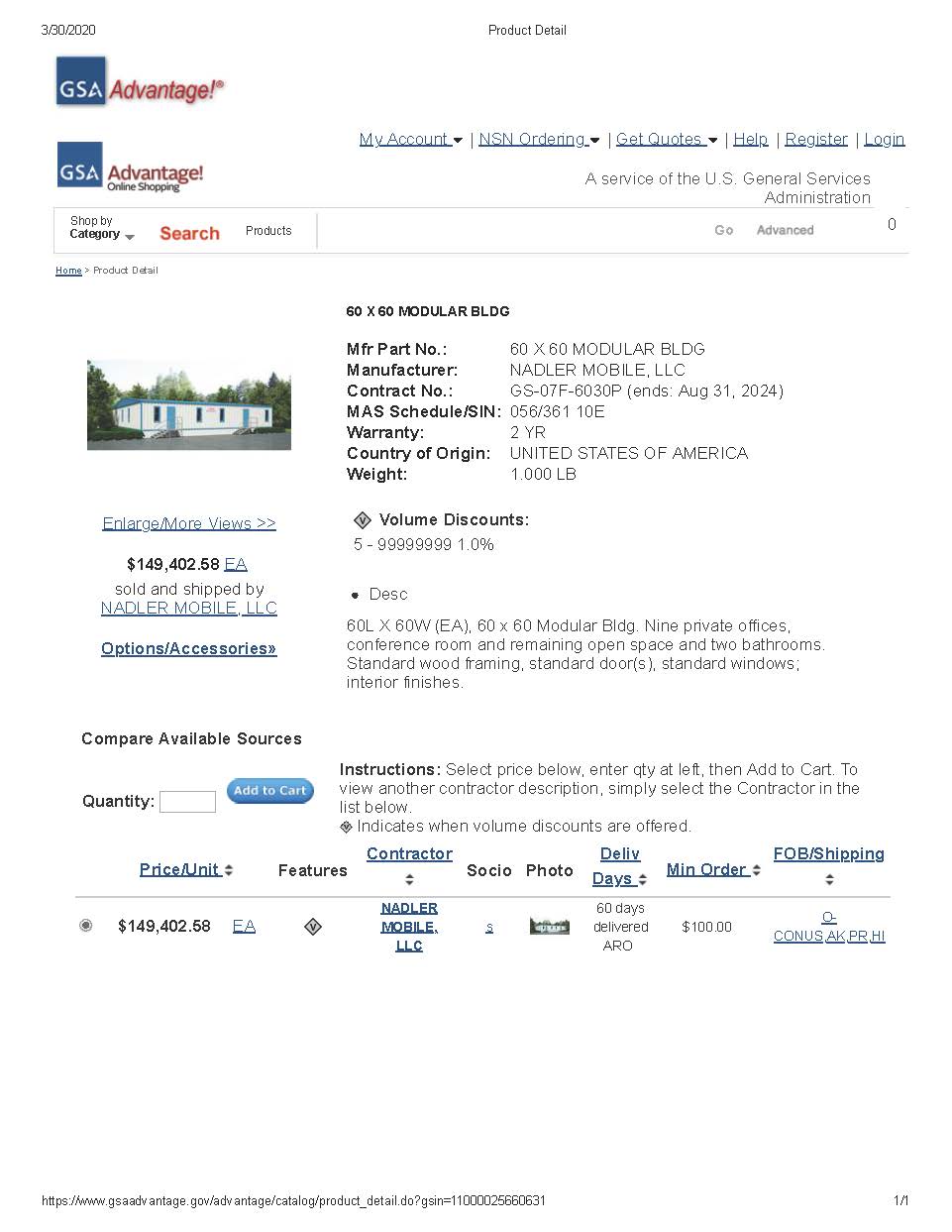


Figure 2: Modular office quote 2.


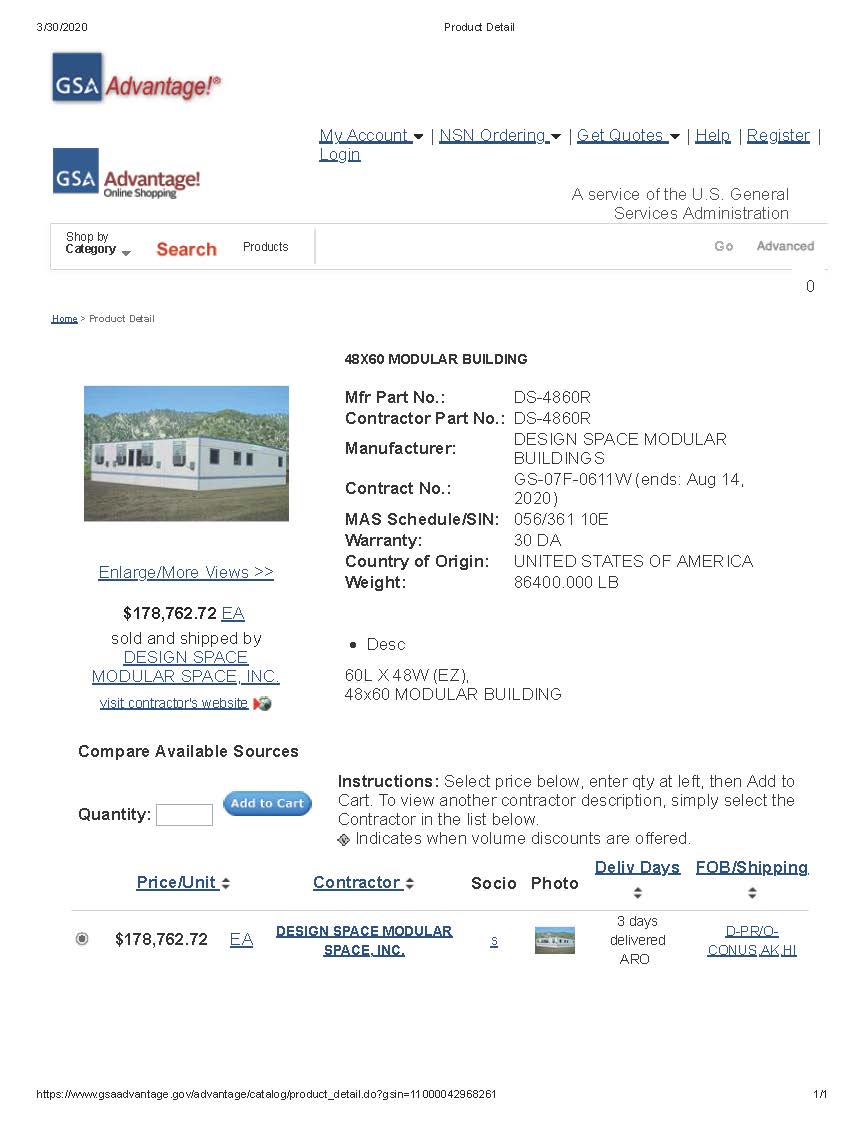


Figure 3: Modular office quote 3.
