# Supplementary material for: A mass rearing cost calculator for the control of Culex quinquefasciatus in Hawaiʻi using the incompatible insect technique
Source: Parasit Vectors. 2022 Dec 5;15:453. doi: 10.1186/s13071-022-05522-1 (PMC9724328; doi:10.1186/s13071-022-05522-1)
Supplement: Supplementary file 4 — Additional file 4: Example report S1. Release cost estimate for control of Cx. quinquefasciatus within ʻiʻiwi’s home range. [file 13071_2022_5522_MOESM4_ESM.docx]

Supplemental Materials Section 3: *Culex quinquefasciatus* IIT/SIT production needs and cost estimate calculator for control within ʻIʻiwi’s home range - Example Report

Adam E. Vorsino, Ph. D.

Ecologist

USFWS, Pacific Islands Fish and Wildlife Office

Email - [Adam_Vorsino@fws.gov](mailto:Adam_Vorsino@fws.gov)

30 August, 2022

# Overview

This report outlines the methods used in the coded analysis (see Supplemental Materials Section 1) to infer mosquito rearing/infrastructure costs associated with controlling mosquitoes at various densities. The information and criteria regarding rearing needs used in this code was provided by The Xi lab at Michigan State University (Dr. Zhiyong Xi *pers. comm*). The output of the code is a table of costs for mosquito control using the SIT/IIT approach (see Zheng et al. (2019)) as applied to the theorized home range of ʻiʻiwi (*Drepanis coccinea*) as estimated elsewhere from the known distribution of ʻōhiʻa lehua (*Metrosideros polymorpha*) forests above 3,500 feet on Kauai, Maui and Hawaiʻi (*Jay Nelson, pers. comm.*). Here the ʻiʻiwi home range area is compared with mosquito densities found in similar sites on the island of Hawaiʻi described in Samuel et al. (2011). The inputs can be modified to reflect other sites. Though it was developed for *Culex quinquefasciatus* infrastructure needs, it is relatively species independent and can be used for other Culicid control efforts (e.g. *Aedes* sp.).

# Methods

Along with information related to the rearing of approximately 1.5 million IIT/SIT derived male mosquitoes (Zheng et al. 2019), Hawaiʻi specific infrastructure, utility and personnel costs were approximated and used in this analysis. All compiled information was used here to assess Infrastructure costs associated with developing a *C. quinquefasciatus* rearing facility at a capacity needed to control known densities in an area the approximate size of the ʻiʻiwi home range. The area can be modified to reflect different sites.

    Table 1 lists the variables used in the code to itemize the infrastructure costs and a description of each variable. Variables to determine infrastructure costs were partitioned into three main types: those variables associated with ecology of Hawaiʻi and the Culicid (*Mosquito and Site Information*), those itemizing the basic infrastructure costs (*Basic Infrastructure Costs*), and those associated with position and personnel expenditures (*Position and Personnel Costs*).

**Table 1:** Variable names and descriptions used in the R code to derive an estimate of infrastructure and personnel costs.

| **Coded Variable** | **Variable Description** |
| --- | --- |
| Mosquito and Site Information | |
| MaunaLoa.Mos.Sites | Sites from Samuel et al. (2011) in which estimates of density were defined |
| MaunaLoa.Mos.Density.km | Mosquito Densities (individuals/sqr. km.) at the various sites from Samuel et al (2011). |
| MaunaLoa.Elev.m | Approximate elevation of sites outlined in the *MaunaLoa.Mos.Sites* input in meters |
| Iiwi.Area.ToCntrl.km | Approx. home range of ʻIʻiwi in square kilometers |
| FemalePercent | The female percent of the sex ratio. A value of 50 indicates an equal sex ratio. |
| Overflooding_Multiplier | Multiplier to derive the overflooding ratio needed for an effective control strategy. A value of 10 indicates that to suppress the wild-type population a successful program needs 10x the number of SIT/IIT laboratory derived Culicid. |
| Basic Infrastructure Costs | |
| Year1.Only.Items | Infrastructure items needed in year 1 for rearing approx. 1.5 million IIT/SIT male Culicid, not including personnel |
| Year1.Only.Costs | Approx. costs of *Year1.Only.Items* for rearing 1.5 million IIT/SIT male Culicid |
| Electricity | Yearly electricity costs to rear approx. 1.5 million IIT/SIT males |
| LaboratorySpace | Cost of a laboratory space needed for rearing approx. 1.5 million IIT/SIT males |
| AllOtherYear.Items | Misc PCR/Lab/Field Supplies for items used in each year of the control application (e.g blood) |
| AllOtherYear.Costs | Approx. costs of *AllOtherYear.Items* for items used in each year of the control application |
| Position and Personnel Costs | |
| Personnel.Des | Types of technician positions to be funded |
| Wage.Mass.Rearing | Average hourly wage for the Mass Rearing Technician position |
| Wage.Quality.Control | Average hourly wage for the Quality Control position |
| HoursPerYear | Yearly hours for each position |
| Fringe | Percent Fringe costs for full time employees |

## Biological/Ecological Characteristics of *C. quinquefasciatus* used for the analysis

As noted in Table 1, mosquito densities were derived from Samuel et al. (2011) (See Appendix B Table 1B in Samuel et al. (2011)) and defined in that paper as the number of individuals per kilometer^2^ for each site. As *C. quinquefasciatus* densities are known to vary based on temperature (Samuel et al. 2011), and elevation and can be used as an imperfect proxy for the variance in temperature between sites, infrastructure costs were determined for all densities of *C. quinquefasciatus* at each elevation in which they were present. Conducting the analysis in this way allows the user of the information to better estimate site and cost variance, and ensure that the calculator was robust to changes in sex ratio.

    For most analyses conducted in this assessment the male to female sex ratio was maintained at 1:1 (equal sex ratio) as it has been observed in the lab. As a test of how robust the assessment is to variation in the sex ratio parameter, the default equal sex ratio was compared to an assessment conducted with a female biased sex ratio. Sex ratios commonly seen in the literature for *C. quinquefasciatus* (or other Culicids) vary (Suleman 1982; Medeiros et al. 2017; David, Ribeiro, and de Freitas 2012), but are within the realm of both ratios used. While maintaining all infrastructure, personnel and density costs, a comparison between an equal (1:1) and female biased sex ratio (70% female, or 0.43:1 male:female ratio, which is the converse of that from David, Ribeiro, and de Freitas (2012)) was conducted to illustrate the variance in cost associated with this variable. In a rearing facility operations are modified such that sex ratio’s are close to equal.

## Infrastructure Costs

As noted above, most year 1 and subsequent year infrastructure costs were estimated using information published (as an average cost/km^2^) in Zheng et al. (2019). One large purchase that was not itemized by these estimates was the Arthropod Containment Level 2 (ACL-2) (Benedict et al. 2018) rearing space necessary to rear approx. 1.5 million male Culicids. It was determined that at maximum a facility must be ~300-500m^2^ (enough to produce 500k - 1 million male SIT/IIT Culicid) to be cost effective, and allow for future scaling efforts. Therefore a 800m^2^ ACL-2 facility (Table 2: *LaboratorySpace*) would be of sufficient size to rear approx. 1.5 million male *C. quinquefasciatus*. The cost of an 800m^2^ facility was estimated using three converted (from ft^2^ to m^2^) median cost quotes (see Supplemental Materials Section 2), as defined per m^2^, and multiplied by the minimum size of a facility (800m^2^). Modification of any modular facility to be ACL-2 compliant is another large cost that must be accounted for (Benedict et al. 2018). Here we infer the cost for an ACL-2 space necessary to rear approx. 1.5 million male Culicid per week (Table 2). The cost of each infrastructure item could vary from this estimate depending on the company used, the facility type, previous ownership (versus new) etc.

    Another high but potentially optional item (if only using IIT) in the year 1 costs is the irradiator used in IIT/SIT to make the females infertile at such low doses as to not affect male fitness (Zheng et al. 2019). If the release program is able to use a machine learning/artificial intelligence adult sex selection discriminator, such as that being developed by Verily Life Sciences (Ovadia et al. 2017) (or decides not to use one at all) these costs may vary. It is important to note that at this point the cost of the irradiator may be lower than the cost of the Verily technology, but that may change. In this report the calculator was run with and without the irradiator costs to illustrate the cost variance of the irradiator (see Tables 1 & 2).

    The most significant perpetual costs (year 1 and beyond) are those associated with rearing and quality control personnel (Table 1 & 2). To rear approx. 1.5 million adult male Culicid every week, eight rearing and three quality control technicians must be fully funded for the extent of the work year (260 days, 8 hours/day, see Table 2). In the calculator presented here the default cost per hour of these different positions was higher for the quality control position as it is primarily managerial (Table 2). These costs are likely on the low end of the wage spectrum for these position types and should be modified as appropriate.

**Table 2:** Variable names and values used in the R code to derive an estimate of infrastructure and personnel costs.

| **Coded Variable** | **Default Variable Value** |
| --- | --- |
| Mosquito and Site Information | |
| MaunaLoa.Mos.Sites | Malama Ki; Nanawale; Bryson's; Waiakea; Cooper; Crater; Pu'u |
| MaunaLoa.Mos.Density.km | 4,546; 78,547; 14,597; 29,001; 27,615; 1,637; 618 |
| MaunaLoa.Elev.m | 25; 36; 314; 885; 1,024; 1,177; 1,247 |
| Iiwi.Area.ToCntrl.km | 1200 kilometers squared |
| FemalePercent | 50% (Assumes an equal sex ratio) |
| Overflooding_Multiplier | 10 (10:1 overflooding ratios are commonly used for a control efficacy of 99% (Zheng et al. 2019; Kandul et al. 2019)) |
| Basic Infrastructure Costs | |
| Year1.Only.Items | ACL2 modification; Irradiator; Mosquito.Sex.Sorters (6); larvae Rearing Units (5); adult cages (100); ovitraps (300); BG traps (50); PCR Machine |
| Year1.Only.Costs | $800,000; $250,000; $6,900; $134,500; $11,040; $2,400; $7,500; $47,000 |
| Electricity | $2,000.00 x 12 |
| LaboratorySpace | median price of a 800 meter squared modular facility (see Supp. Mat. Section 2 for price quotes) |
| AllOtherYear.Items | PCR Buffers, reagents, primers, Taq, Misc. Equipment, Misc. Field Supplies. |
| AllOtherYear.Costs | $30,000 |
| Position and Personnel Costs | |
| Personnel.Des | Mass Rearing; Quality Control |
| Wage.Mass.Rearing | $20.00 |
| Wage.Quality.Control | $25.00 |
| HoursPerYear | 260 * 8 |
| Fringe | Research Corporation of the University of Hawaiʻi Fringe/Indirect is set at 61.56% for 2018. |

## Ratio Calculation

It is assumed here that each [larval rearing unit](https://www.vienna-scientific.com/products/tray-and-rack-systems/) can rear ~ 1 million Culicidae/week (Table 1) (Balestrino, Benedict, and Gilles 2012; Zhang et al. 2017). In this calculator the larval rearing units are treated as the primary delimiter defining the production scale of a Culicid mass rearing facility. In other words, each incremental increase in the number of larval rearing units necessitates a certain amount of space (for laboratory work, adult rearing, sex-sorting, office space etc.), positions and other costs outlined in Table 1. Under this assumption, to house 1 rearing unit associated staff etc., a facility would need at maximum a 300-500m^2^ laboratory space, and to house 5 rearing units and the associated staff etc., it is assumed that a facility would need at maximum a 800m^2^ laboratory space. The rate of increase used for each assessment is supplied in each table. These rates can be thought of as both the number of larval rearing units needed as well as the rate of increase for all other items (space, positions etc.) associated with rearing the necessary density of male Culicidae (Table 2). These rates are rounded up to the nearest whole unit, from two significant digits of the proportion of wild-type males to laboratory males needed. Conducting the assessment in this way ensures the production capacity needed for the successful implementation of an IIT or IIT/SIT control program.

# Results

In this calculator the rate of increase, and thus the proportion of resources needed, is determined by the number of larval rearing units required to reach the necessary density of male Culicidae/*C. quinquefasciatus*. The rate may vary based on certain assumptions associated with the proportion of wild-type males to the number of IIT/SIT males needed to control those wild type individuals. Those assumptions that may vary this rate of increase are primarily associated with the capacity of the larval rearing units (Table 1) or the biology/ecology of *C.quinquefasciatus* (see Table 2). The differences between Table 3 and Table 4 show how these rates may vary the cost of control given certain assumptions regarding the sex ratio of the laboratory reared individuals. Specifically, Table 3 shows the rates needed when using a female biased sex ratio as applied to an elevation gradient in *C. quinquefasciatus* densities for an SIT/IIT program the size of ʻiʻiwi’s home range, and Table 4 applies all default assumptions.

**Table 3:** IIT/SIT male mass release production and costs for a 1200 km² area. In the table a rate of 1 is equivalent to the production of ≤ 1 million IIT/SIT Culicidae using the 0.43:1 female biased sex ratio. Those areas highlighted in red have elevations relatively equivalent to the minimum elevation ʻiʻiwi’s home range. Please note: this does not include costs associated with community outreach or field releases.

| **Site** | **Elevation (m)^1^** | **Wild Type Males** | **IIT:Wild Type Males (10:1)^2^** | **Rate Used** | **First Year Cost ($)^3^** | **Subsequent Year Costs ($)** |
| --- | --- | --- | --- | --- | --- | --- |
| Malama Ki | 25 | 2,727,600 | 27,276,000 | 91 | $40,543,133.00 | $17,102,636.00 |
| Nanawale | 36 | 47,128,200 | 471,282,000 | 1,600 | $708,039,583.00 | $300,705,690.00 |
| Bryson's | 314 | 8,758,200 | 87,582,000 | 290 | $128,536,862.00 | $54,502,906.00 |
| Waiakea | 885 | 17,400,600 | 174,006,000 | 580 | $256,823,724.00 | $109,005,812.00 |
| Cooper | 1,024 | 16,569,000 | 165,690,000 | 550 | $243,552,669.00 | $103,367,581.00 |
| *Crater* | *1,177* | *982,200* | *9,822,000* | *33* | *$14,866,960.00* | *$6,202,055.00* |
| *Pu'u* | *1,247* | *370,800* | *3,708,000* | *12* | *$5,586,622.00* | *$2,255,293.00* |
| *^1^Kokee State Park Visitors Center Elevation is ~1,115 meters; Alakai Swamp Elevation is ~1,219-1,402 meters.* | | | | | | |
| *^2^Alakai Wilderness Reserve with a 2 kilometer buffer has a combined area of 117 kilometers squared.* | | | | | | |
| *^3^This does not include mosquito dispersal/application costs.* | | | | | | |

When modifying the default assumptions (Table 2) to use a female biased sex ratio (Table 3) the overall costs are substantially greater then that of the analysis conducted with the default equal sex ratio (Table 4). Though the production costs calculated using this approach are relatively robust to small variances in the sex ratio of the laboratory reared individuals, the buffering effect that associates linking rate to rearing units is removed when projected for large quantities.

**Table 4:** IIT/SIT male mass release production and costs for a 1200 km² area. In the table a rate of 1 is equivalent to the production of ≤ 1 million IIT/SIT Culicidae using equal sex ratio. Those areas highlighted in red have elevations relatively equivalent to the minimum elevation ʻiʻiwi’s home range. Please note: this does not include costs associated with community outreach or field releases.

| **Site** | **Elevation (m)^1^** | **Wild Type Males** | **IIT:Wild Type Males (10:1)^2^** | **Rate Used** | **First Year Cost ($)^3^** | **Subsequent Year Costs ($)** |
| --- | --- | --- | --- | --- | --- | --- |
| Malama Ki | 25 | 2,727,600 | 27,276,000 | 91 | $40,543,133.00 | $17,102,636.00 |
| Nanawale | 36 | 47,128,200 | 471,282,000 | 1,600 | $708,039,583.00 | $300,705,690.00 |
| Bryson's | 314 | 8,758,200 | 87,582,000 | 290 | $128,536,862.00 | $54,502,906.00 |
| Waiakea | 885 | 17,400,600 | 174,006,000 | 580 | $256,823,724.00 | $109,005,812.00 |
| Cooper | 1,024 | 16,569,000 | 165,690,000 | 550 | $243,552,669.00 | $103,367,581.00 |
| *Crater* | *1,177* | *982,200* | *9,822,000* | *33* | *$14,866,960.00* | *$6,202,055.00* |
| *Pu'u* | *1,247* | *370,800* | *3,708,000* | *12* | *$5,586,622.00* | *$2,255,293.00* |
| *^1^Kokee State Park Visitors Center Elevation is ~1,115 meters; Alakai Swamp Elevation is ~1,219-1,402 meters.* | | | | | | |
| *^2^Alakai Wilderness Reserve with a 2 kilometer buffer has a combined area of 117 kilometers squared.* | | | | | | |
| *^3^This does not include mosquito dispersal/application costs.* | | | | | | |

As noted in the methods section, two other costs that should be examined more closely are the costs of the mobile laboratory, and the irradiator (Table 2). Both the mobile laboratory and irradiator are significant to the overall effort in that they provide capacity for the effort (e.g. lab space) and confidence in the continued efficacy of the overall approach (e.g. irradiator)(Zheng et al. 2019), but these costs could potentially be offset by matching efforts from other institutions/organizations. Table 5 shows the infrastructure costs for the IIT/SIT effort without the cost of a rearing facility.

**Table 5:** IIT/SIT male mass release production and costs for a 1200 km² area. In the table a rate of 1 is equivalent to the production of ≤ 1 million IIT/SIT Culicidae. The mobile laboratory costs have been removed from this table. Those areas highlighted in red have elevations relatively equivalent to the minimum elevation ʻiʻiwi’s home range. Please note: this does not include costs associated with community outreach or field releases.

| **Site** | **Elevation (m)^1^** | **Wild Type Males** | **IIT:Wild Type Males (10:1)^2^** | **Rate Used** | **First Year Cost ($)^3^** | **Subsequent Year Costs ($)** |
| --- | --- | --- | --- | --- | --- | --- |
| Malama Ki | 25 | 2,727,600 | 27,276,000 | 91 | $34,013,024.00 | $17,102,636.00 |
| Nanawale | 36 | 47,128,200 | 471,282,000 | 1,600 | $593,224,490.00 | $300,705,690.00 |
| Bryson's | 314 | 8,758,200 | 87,582,000 | 290 | $107,726,626.00 | $54,502,906.00 |
| Waiakea | 885 | 17,400,600 | 174,006,000 | 580 | $215,203,252.00 | $109,005,812.00 |
| Cooper | 1,024 | 16,569,000 | 165,690,000 | 550 | $204,084,981.00 | $103,367,581.00 |
| *Crater* | *1,177* | *982,200* | *9,822,000* | *33* | *$12,498,899.00* | *$6,202,055.00* |
| *Pu'u* | *1,247* | *370,800* | *3,708,000* | *12* | *$4,725,509.00* | *$2,255,293.00* |
| *^1^Kokee State Park Visitors Center Elevation is ~1,115 meters; Alakai Swamp Elevation is ~1,219-1,402 meters.* | | | | | | |
| *^2^Alakai Wilderness Reserve with a 2 kilometer buffer has a combined area of 117 kilometers squared.* | | | | | | |
| *^3^This does not include mosquito dispersal/application costs.* | | | | | | |

As noted in Table 2, the cost of the irradiator is a significant investment. Table 6 shows the cost of the irradiator with all other default infrastructure costs. Something that should be noted when removing both rearing facility and irradiator costs (Tables 5 & 6) is that the rate of infrastructure increase (i.e. number of larval rearing units and associated costs needed) is the same, in fact this is true for all assessments with default assumptions (Tables 4-6). This is because the rate acts as a proportion from which these unit costs are estimated, and is not influenced by these costs.

**Table 6:** IIT/SIT male mass release production and costs for a 1200 km² area. In the table a rate of 1 is equivalent to the production of ≤ 1 million IIT/SIT Culicidae. The irradiator costs have been removed from this table. Those areas highlighted in red have elevations relatively equivalent to the minimum elevation ʻiʻiwi’s home range. Please note: this does not include costs associated with community outreach or field releases.

| **Site** | **Elevation (m)^1^** | **Wild Type Males** | **IIT:Wild Type Males (10:1)^2^** | **Rate Used** | **First Year Cost ($)^3^** | **Subsequent Year Costs ($)** |
| --- | --- | --- | --- | --- | --- | --- |
| Malama Ki | 25 | 2,727,600 | 27,276,000 | 91 | $40,293,133.00 | $17,102,636.00 |
| Nanawale | 36 | 47,128,200 | 471,282,000 | 1,600 | $707,789,583.00 | $300,705,690.00 |
| Bryson's | 314 | 8,758,200 | 87,582,000 | 290 | $128,286,862.00 | $54,502,906.00 |
| Waiakea | 885 | 17,400,600 | 174,006,000 | 580 | $256,573,724.00 | $109,005,812.00 |
| Cooper | 1,024 | 16,569,000 | 165,690,000 | 550 | $243,302,669.00 | $103,367,581.00 |
| *Crater* | *1,177* | *982,200* | *9,822,000* | *33* | *$14,616,960.00* | *$6,202,055.00* |
| *Pu'u* | *1,247* | *370,800* | *3,708,000* | *12* | *$5,336,622.00* | *$2,255,293.00* |
| *^1^Kokee State Park Visitors Center Elevation is ~1,115 meters; Alakai Swamp Elevation is ~1,219-1,402 meters.* | | | | | | |
| *^2^Alakai Wilderness Reserve with a 2 kilometer buffer has a combined area of 117 kilometers squared.* | | | | | | |
| *^3^This does not include mosquito dispersal/application costs.* | | | | | | |

# References

Balestrino, F., M. Q. Benedict, and J. R. L. Gilles. 2012. “A New Larval Tray and Rack System for Improved Mosquito Mass Rearing.” *Journal of Medical Entomology* 49 (3): 595–605. <https://doi.org/10.1603/ME11188>.

Benedict, Mark Q., Austin Burt, Margareth L. Capurro, Paul De Barro, Alfred M. Handler, Keith R. Hayes, John M. Marshall, Walter J. Tabachnick, and Zach N. Adelman. 2018. “Recommendations for Laboratory Containment and Management of Gene Drive Systems in Arthropods.” *Vector-Borne and Zoonotic Diseases* 18 (1): 2–13. <https://doi.org/10.1089/vbz.2017.2121>.

David, Mariana Rocha, Gabriel Sylvestre Ribeiro, and Rafael Maciel de Freitas. 2012. “Bionomics of *Culex Quinquefasciatus* Within Urban Areas of Rio de Janeiro, Southeastern Brazil.” *Revista de Saúde Pública* 46 (5): 858–65. <https://doi.org/10.1590/S0034-89102012000500013>.

Kandul, Nikolay P., Junru Liu, Hector M. Sanchez C, Sean L. Wu, John M. Marshall, and Omar S. Akbari. 2019. “Transforming Insect Population Control with Precision Guided Sterile Males with Demonstration in Flies.” *Nature Communications* 10 (1): 1–12. <https://doi.org/10.1038/s41467-018-07964-7>.

Medeiros, Matthew C. I., Emily C. Boothe, E. Brendan Roark, and Gabriel L. Hamer. 2017. “Dispersal of Male and Female *Culex Quinquefasciatus* and *Aedes Albopictus* Mosquitoes Using Stable Isotope Enrichment.” *PLoS Neglected Tropical Diseases* 11 (1). <https://doi.org/10.1371/journal.pntd.0005347>.

Ovadia, Yaniv, Yoni Halpern, Dilip Krishnan, Josh Livni, Daniel Newburger, Ryan Poplin, Tiantian Zha, and D. Sculley. 2017. “Learning to Count Mosquitoes for the Sterile Insect Technique.” In *Proceedings of the 23rd ACM SIGKDD International Conference on Knowledge Discovery and Data Mining*, 1943–49. KDD ’17. Halifax, NS, Canada: Association for Computing Machinery. <https://doi.org/10.1145/3097983.3098204>.

Samuel, Michael D., Peter HF Hobbelen, Francisco DeCastro, Jorge A. Ahumada, Dennis A. LaPointe, Carter T. Atkinson, Bethany L. Woodworth, Patrick J. Hart, and David C. Duffy. 2011. “The Dynamics, Transmission, and Population Impacts of Avian Malaria in Native Hawaiian Birds: A Modeling Approach.” *Ecological Applications* 21 (8): 2960–73.

Suleman, M. 1982. “The Effects of Intraspecific Competition for Food and Space on the Larval Development of *Culex Quinquefasciatus.*” *Mosquito News* 42 (3): 347–56.

Zhang, Dongjing, Meichun Zhang, Yu Wu, Jeremie R. L. Gilles, Hanano Yamada, Zhongdao Wu, Zhiyong Xi, and Xiaoying Zheng. 2017. “Establishment of a Medium-Scale Mosquito Facility: Optimization of the Larval Mass-Rearing Unit for *Aedes Albopictus* *(Diptera:* *Culicidae)*.” *Parasites & Vectors* 10 (1): 569. <https://doi.org/10.1186/s13071-017-2511-z>.

Zheng, Xiaoying, Dongjing Zhang, Yongjun Li, Cui Yang, Yu Wu, Xiao Liang, Yongkang Liang, et al. 2019. “Incompatible and Sterile Insect Techniques Combined Eliminate Mosquitoes.” *Nature* 572 (7767): 56–61. <https://doi.org/10.1038/s41586-019-1407-9>.
