# Supplementary material for: A mass rearing cost calculator for the control of Culex quinquefasciatus in Hawaiʻi using the incompatible insect technique
Source: Parasit Vectors. 2022 Dec 5;15:453. doi: 10.1186/s13071-022-05522-1 (PMC9724328; doi:10.1186/s13071-022-05522-1)
Supplement: Supplementary file 5 — Additional file 5: Example Report S2. Release cost estimate for control of Ae. aegypti on the island of Hawaiʻi. [file 13071_2022_5522_MOESM5_ESM.docx]

Supplemental Materials Section 4: *Aedes aegypti* IIT/SIT production needs and cost estimate calculator - Example Report

Adam E. Vorsino, Ph. D.

Ecologist

USFWS, Pacific Islands Fish and Wildlife Office

Email - [Adam_Vorsino@fws.gov](mailto:Adam_Vorsino@fws.gov)

30 August, 2022

# Overview

This report outlines the methods used in the coded analysis (see [Supp. Mat. Section 1](file:///C:\Users\avorsino\OneDrive%20-%20DOI\Desktop\RCode\GitHub\Mosquito_Release_Cost\Supplemental%20Materials%20Section%201:%20Code%20used%20to%20infer%20instrastructure%20costs)) to infer mosquito rearing/infrastructure costs associated with controlling mosquitoes at various densities. The information and criteria regarding rearing needs used in this code was provided by The Xi lab at Michigan State University (Dr. Zhiyong Xi *pers. comm*). The output of the code is a table of costs for mosquito control using the SIT/IIT approach (see (**zheng19?**)) as applied to an area suggested by the Hawaiʻi Department of Health (HDoH) to be the pimrary area on the Island of Hawaiʻi in which *Aedes aegypti* occupy. Mosquito densities were calculated based on [Biogents Sentinel](https://www.myadapco.com/product/bg-sentinel-2-trap/) (BG) trap collections conducted by the HDoH, and estimates of flight distance. Though initially the calculator described here was developed for *Culex quinquefasciatus* infrastructure needs, it is relatively species independent and so has been modified to reflect *A. aegypti* infrastructure costs.

# Methods

Along with information provided by Dr. Zhiyong Xi for the rearing of approximately 1.5 million Insect Incompatibility Technique (IIT) /Sterile Insect Technique (SIT) derived male mosquitoes (**zheng19?**), Hawaiʻi specific infrastructure, utility and personnel costs were approximated and used in this analysis. All compiled information was used here to assess Infrastructure costs associated with developing a Culicid rearing facility at a capacity needed to control estimated *A. aegypti* densities for a 1000km^2^ site.

Table 1 lists the variables used in the code to itemize the infrastructure costs and a description of each variable. Variables to determine infrastructure costs naturally partitioned into three main types: Those associated with ecology of Hawaiʻi and the Culicid (*Mosquito and Site Information*), those variables itemizing the basic infrastructure costs (*Basic Infrastructure Costs*), and those variables associated with position and personnel expenditures (*Position and Personnel Costs*).

**Table 1:** Variable names and descriptions used in the R code to derive an estimate of infrastructure and personnel costs.

| **CODED VARIABLE** | **VARIABLE DESCRIPTION** |
| --- | --- |
| *MOSQUITO AND SITE INFORMATION* | |
| *maxRadius* | Flight Distance (in meters) of the Culicid. |
| *MaxCollection* | Average or Maximum Collection amount from BG traps of Culicids on the Island of Hawai`r knitr::asis_output("ʻ")`i by the Hawaiʻi Department of Health |
| *Aedes.Mos.Density.km* | Mosquito Densities (individuals/sqr. km.) were by estimated by dividing the maximum number of collected Culicids in BG traps by the collection area. The flight distance (maxRadius) was used as the radius to determine the area of collection site   (i.e. area of a circle). |
| *Total.Area.ToCntrl.km* | Approx. area the Culicid occupies on the Island of Hawaiʻi |
| *FemalePercent* | The female percent of the sex ratio. A value of 50 indicates an equal sex ratio. |
| *Overflooding_Multiplier* | Multiplier to derive the overflooding ratio needed for an effective control strategy. A value of 10 indicates that to suppress the wild-type population a successful program needs 10x the number of SIT/IIT laboratory derived Culicidae. |
| *BASIC INFRASTRUCTURE COSTS* | |
| *Year1.Only.Items* | Infrastructure items needed in year 1 for rearing ≤ 1.5 million IIT/SIT male Culicid, not including personnel |
| *Year1.Only.Costs* | Approx. costs of *Year1.Only.Items* for rearing ≤ 1.5 million IIT/SIT male Culicid |
| *Electricity* | Yearly electricity costs to rear ≤ 1.5 million IIT/SIT males |
| *LaboratorySpace* | Cost of a 800 meter squared laboratory space as needed for rearing ≤ 1.5 million IIT/SIT males |
| *AllOtherYear.Items* | Misc PCR/Lab/Feild Supplies for items used in each year of the control application |
| *AllOtherYear.Costs* | Approx. costs of *AllOtherYear.Items* for items used in each year of the control application |
| *POSITION AND PERSONNEL COSTS* | |
| *Personnel.Des* | Types of technician positions to be funded |
| *Wage.Mass.Rearing* | Average hourly wage for the Mass Rearing Technician position |
| *Wage.Quality.Control* | Average hourly wage for the Quality Control position |
| *HoursPerYear* | Yearly hours for each position |
| *Fringe* | Percent Fringe costs for full time employees |

## Biological/Ecological Characteristics of *A. aegypti* used for the analysis

As noted in Table 1, mosquito densities were estimated using a series of assumptions, these were necessary as the author is not aware of any current density estimates for *A. aegypti* in Hawaiʻi. The first assumption necessary was that a conservative estimate of density could be calculated using BG trap collections by using known *A. aegypti* flight distances in combination with the maximum number of *A. aegypti* collected on the Island of Hawaiʻi by HDoH. Flight distances were sequentially increased from 50 to 400 meters to show how its variance may affect infrastructure costs. The flight distances used fell within those reported by Verdonschot and Besse-Lototskaya (2014), and a lifetime flight distance estimated by the World Health Organization (WHO 2020) for *A. aegypti*. These flight distances were used as the radius of collection for a BG trap and from this the area of collection was calculated. To estimate density the maximum number of BG trap collections reported in Hawaiʻi (181 *A. aegypti*, Lincoln Wells, *pers. comm.* ) was used as the numerator (with the area of collection as the denominator) for each density estimate. This was then converted into km^2^ and multiplied by 1000 to get the total estimated wildtype population under each scenario on the Island of Hawaiʻi. Conducting the analysis in this way allows the user of the information to better estimate site and cost variance. This assumption assumes equivalence of density throughout the treatment area.

For most analyses conducted in this assessment the male to female sex ratio was maintained at 1:1 (equal sex ratio) as it has been observed in the lab. As a test of how robust the assessment is to variation in the sex ratio parameter, the default equal sex ratio was compared to an assessment conducted with a female biased sex ratio (0.43:1). Sex ratios commonly seen in the literature for *A. aegypti* (or Culicids) can vary by habitat (Qureshi et al. 2019), but are within the realm of both ratios used. While maintaining all infrastructure, personnel and density costs, a comparison between an equal and female biased sex ratio was conducted to illustrate the variance in cost associated with this variable when applied to the laboratory reared individuals. In a rearing facility operations are modified such that sex ratio’s are close to equal.

## Infrastructure Costs

As noted above, most year 1 and subsequent year infrastructure costs were estimated using information provided by Dr. Xi and published (as an average cost/km^2^) in (**zheng19?**). One large purchase that was not itemized by Dr. Xi’s estimates was the Arthropod Containment Level 2 (ACL-2) (Benedict et al. 2018) rearing space necessary to rear approximately 1.5 million male Culicids. From subsequent information provided by Dr. Xi it was estimated that at minimum a facility must be ~300-500m^2^ (enough to produce 500k - 1 million male SIT/IIT Culicid) to be cost effective, and allow for future scaling efforts. With this estimate, it was estimated that an 800m^2^ ACL-2 facility (Table 2: *LaboratorySpace*) would be a sufficient size to rear approx. 1.5 million male Culicids. The cost of an 800 m^2^ facility was estimated using three converted (from ft^2^ to m^2^) median cost quotes (see [Supp. Mat. Section 2][Supplemental Materials Section 2: Quotes used to derive facility per meter cost estimate]), as defined per m^2^, and multiplying by the size of the facility (800 m^2^). Modification of any modular facility to be ACL-2 compliant is another large cost that must be accounted for (Benedict et al. 2018). Here we infer the cost for an ACL-2 space necessary to rear approx. 1.5 million male Culicid per week (Table 2). The cost of each infrastructure item could vary from this estimate depending on the company used, the facility type, previous ownership (versus new) etc

Another high but potentially optional item in the year 1 costs is the irradiator used to make the females infertile at such low doses as to not affect male fitness (**zheng19?**). If the release program is able to use a machine learning/artificial intelligence adult sex selection discriminator, such as that being developed by Verily Life Sciences (Ovadia et al. 2017) (or decides not to use one at all) these costs may vary. It is important to note that the cost of the irradiator *may be lower* (Dr. Zhiyong Xi, *pers. comm*) then the cost of the Verily technology. In this report the calculator was run with and without the irradiator costs to illustrate the cost variance of the irradiator (see Tables 1 & 2), .

The most significant perpetual costs (year 1 and beyond) are those associated with rearing and quality control personnel (Table 1 & 2). To rear $\leq$ 1.5 million adult male Culicid every week, eight rearing and three quality control technicians must be fully funded for the extent of the work year (260 days, 8 hours/day, see Table 2). In the calculator presented here the default cost per hour of these different positions was higher for the quality control position as it is primarily oversite based (Table 2). These costs are likely on the low end of the wage spectrum for these position types.

**Table 2:** Variable names and values used in the R code to derive an estimate of infrastructure and personnel costs.

| **CODED VARIABLE** | **DEFAULT VARIABLE VALUE** |
| --- | --- |
| *MOSQUITO AND SITE INFORMATION* | |
| *maxRadius* | 50; 100; 150; 200; 250; 300; 350; 400: Flight distances (in meters) derived from Verdonschot *et al.* (2014) and the World Health Organization (WHO) |
| *Aedes.Mos.Density.km* | ((MaxCollection/(pi*maxRadiuss^2))*1000000) |
| *Total.Area.ToCntrl.km* | 1000 kilometers squared |
| *FemalePercent* | 50% (Assumes equal sex ratio) |
| *Overflooding_Multiplier* | 10 (10:1 overflooding ratios are commonly used for a control efficacy of 99% (Zheng et al. 2019; Kandul et al. 2019)) |
| *BASIC INFRASTRUCTURE COSTS* | |
| *Year1.Only.Items* | ACL2 modification; Irradiator; Mosquito.Sex.Sorters (6); larvae Rearing Units (5); adult cages (100); ovitraps (300); BG traps (50); PCR Machine |
| *Year1.Only.Costs* | $800,000; $250,000; $6,900; $134,500; $11,040; $2,400; $7,500; $47,000 |
| *Electricity* | $2,000.00 x 12 |
| *LaboratorySpace* | median price of a 800 meter squared modular facility (see Supp. Mat. Section 2 for price quotes) |
| *AllOtherYear.Items* | PCR Buffers, reagents, primers, Taq, Misc. Equipment, Misc. Feild Supplies. |
| *AllOtherYear.Costs* | $30,000 |
| *POSITION AND PERSONNEL COSTS* | |
| *Personnel.Des* | Mass Rearing; Quality Control |
| *Wage.Mass.Rearing* | $15.00 |
| *Wage.Quality.Control* | $17.00 |
| *HoursPerYear* | 260 * 8 |
| *Fringe* | Research Corporation of the University of Hawaiʻi Fringe/Indirect is set at 61.56% for 2018. |

## Ratio Calculation

It is assumed here that each [larval rearing unit](https://www.vienna-scientific.com/products/tray-and-rack-systems/) can rear ~ 1 million Culicidae/week (Table 1) (Balestrino, Benedict, and Gilles 2012; Zhang et al. 2017). In this calculator the larval rearing units are treated as the primary delimiter defining the production scale of a Culicid mass rearing facility. In other words, each incremental increase in the number of larval rearing units necessitates a certain amount of space, positions and other costs outlined in Table 1. Under this assumption, to house 1 rearing unit associated staff etc., a facility would need at minimum a 300-500m^2^ laboratory space, and to house 5 rearing units and the associated staff etc., it is assumed that a facility would need at minimum a 800m^2^ laboratory space. The rate of increase used for each assessment is supplied in each table. These rates can be thought of as both the number of larval rearing units needed as well as the rate of increase for all other items associated with rearing the necessary density of male Culicidae (Table 2). These rates are rounded up to the nearest whole unit, from two significant digits of the proportion of wild-type males to laboratory males needed. Conducting the assessment in this way ensures the production capacity needed for the successful implementation of an IIT/SIT control program.

# Results

In this calculator the rate of increase, and thus the proportion of resources needed, is determined by the number of larval rearing units required to reach the necessary density of male *A. aegypti*. The rate may vary based on certain assumptions associated with the proportion of wild-type males to the number of IIT or IIT/SIT males needed to control those wild type individuals (overflooding ratio). Those assumptions that may vary this rate of increase are primarily associated with the capacity of the larval rearing units or the biology/ecology of *A. aegypti* (see Table 2). The differences between Table 3 and Table 4 show how these rates may vary the cost of control given certain assumptions regarding the sex ratio of the laboratory reared individuals. Specifically, Table 3 shows the rates needed when using a female biased sex ratio as applied to are applied to a density gradient of *A. aegypti* densities for a 1000 km^2^ treated area

**Table 3:** IIT/SIT male mass release production and costs for a 1000 km² area. In the table a rate of 1 is equivalent to the production of ≤ 1 million IIT/SIT Culicidae using the 0.43:1 female biased sex ratio. Rows higlighted in red denote possible flight distances derived from Verdonschot et al. (2014), and those in blue represent the average distance adults may fly in a lifetime, as derived from the World Health Organization (WHO) estimate.

| **Culicid Density (per square km.)** | **Flight Distance (meters)** | **Wild Type Males** | **IIT:Wild Type Males (10:1)** | **Rate Used** | **First Year Cost ($)^1^** | **Subsequent Year Costs ($)** |
| --- | --- | --- | --- | --- | --- | --- |
| ***23,045.6358*** | 50 | 23,045,636 | 230,456,360 | 770 | $216,412,162.00 | $111,594,038.00 |
| ***5,761.4089*** | 100 | 5,761,409 | 57,614,090 | 190 | $54,038,404.00 | $27,536,191.00 |
| ***2,560.6262*** | *150* | *2,560,626* | *25,606,260* | *85* | *$24,643,154.00* | *$12,318,822.00* |
| ***1,440.3522*** | *200* | *1,440,352* | *14,403,520* | *48* | *$14,287,828.00* | *$6,956,511.00* |
| ***921.8254*** | 250 | 921,825 | 9,218,250 | 31 | $9,531,597.00 | $4,492,747.00 |
| ***640.1565*** | 300 | 640,157 | 6,401,570 | 21 | $6,732,050.00 | $3,043,474.00 |
| ***470.3191*** | 350 | 470,319 | 4,703,190 | 16 | $5,332,276.00 | $2,318,837.00 |
| ***360.0881*** | *400* | *360,088* | *3,600,880* | *12* | *$4,210,957.00* | *$1,739,128.00* |
| *^1^This does not include mosquito dispersal/application costs.* | | | | | | |

When modifying the default assumptions (Table 2) to use a female biased sex ratio (Table 3) the overall costs are substantially greater then that of the analysis conducted with the default equal sex ratio (Table 4). Though the production costs calculated using this approach are relatively robust to small variances in the sex ratio of the laboratory reared individuals, the buffering effect that links rate of increase to rearing units is removed when projected for large quantities.

| **Culicid Density (per square km.)** | **Flight Distance (meters)** | **Wild Type Males** | **IIT:Wild Type Males (10:1)** | **Rate Used** | **First Year Cost ($)^1^** | **Subsequent Year Costs ($)** |
| --- | --- | --- | --- | --- | --- | --- |
| ***23,045.6358*** | 50 | 23,045,636 | 230,456,360 | 460 | $129,626,187.00 | $66,666,568.00 |
| ***5,761.4089*** | 100 | 5,761,409 | 57,614,090 | 120 | $34,441,571.00 | $17,391,279.00 |
| ***2,560.6262*** | *150* | *2,560,626* | *25,606,260* | *51* | *$15,130,693.00* | *$7,391,293.00* |
| ***1,440.3522*** | *200* | *1,440,352* | *14,403,520* | *29* | *$8,967,188.00* | *$4,202,892.00* |
| ***921.8254*** | 250 | 921,825 | 9,218,250 | 18 | $5,889,186.00 | $2,608,692.00 |
| ***640.1565*** | 300 | 640,157 | 6,401,570 | 13 | $4,489,412.00 | $1,884,055.00 |
| ***470.3191*** | 350 | 470,319 | 4,703,190 | 10 | $3,646,548.00 | $1,449,273.00 |
| ***360.0881*** | *400* | *360,088* | *3,600,880* | *8* | *$3,089,638.00* | *$1,159,419.00* |
| *^1^This does not include mosquito dispersal/application costs.* | | | | | | |

As noted in the methods section, two other costs that should be examined more closely are the costs of the mobile laboratory, and the irriadiator (Table 1 & 2). Both the mobile laboratory and irradiator are significant to the overall effort in that they provide capacity for the effort (e.g. lab space) and confidence in the continued efficacy of the overall approach (e.g. irradiator)(**zheng19?**), but these costs could potentially be offset by matching efforts from other institutions/organizations. Table 5 shows the infrastructure costs for the IIT/SIT effort without the cost of a rearing facility.

| **Culicid Density (per square km.)** | **Flight Distance (meters)** | **Wild Type Males** | **IIT:Wild Type Males (10:1)** | **Rate Used** | **First Year Cost ($)^1^** | **Subsequent Year Costs ($)** |
| --- | --- | --- | --- | --- | --- | --- |
| ***23,045.6358*** | 50 | 23,045,636 | 230,456,360 | 460 | $96,616,848.00 | $66,666,568.00 |
| ***5,761.4089*** | 100 | 5,761,409 | 57,614,090 | 120 | $25,830,439.00 | $17,391,279.00 |
| ***2,560.6262*** | *150* | *2,560,626* | *25,606,260* | *51* | *$11,470,961.00* | *$7,391,293.00* |
| ***1,440.3522*** | *200* | *1,440,352* | *14,403,520* | *29* | *$6,886,164.00* | *$4,202,892.00* |
| ***921.8254*** | 250 | 921,825 | 9,218,250 | 18 | $4,597,516.00 | $2,608,692.00 |
| ***640.1565*** | 300 | 640,157 | 6,401,570 | 13 | $3,556,539.00 | $1,884,055.00 |
| ***470.3191*** | 350 | 470,319 | 4,703,190 | 10 | $2,928,953.00 | $1,449,273.00 |
| ***360.0881*** | *400* | *360,088* | *3,600,880* | *8* | *$2,515,563.00* | *$1,159,419.00* |
| *^1^This does not include mosquito dispersal/application costs.* | | | | | | |

As noted in Table 2, the cost of the irradiator is a significant investment. Table 6 shows the cost of the irradiator with all other default infrastructure costs. Something that should be noted when removing both rearing facility and irradiator costs (Tables 5 & 6) is that the rate of infrastructure increase (i.e. number of larval rearing units and associated costs needed) is the same, in fact this is true for all assessments (Tables 3-6). This is because the rate acts as a proportion from which these unit costs are estimated, and is not influenced by these costs.

| **Culicid Density (per square km.)** | **Flight Distance (meters)** | **Wild Type Males** | **IIT:Wild Type Males (10:1)** | **Rate Used** | **First Year Cost ($)^1^** | **Subsequent Year Costs ($)** |
| --- | --- | --- | --- | --- | --- | --- |
| ***23,045.6358*** | 50 | 23,045,636 | 230,456,360 | 460 | $96,616,848.00 | $66,666,568.00 |
| ***5,761.4089*** | 100 | 5,761,409 | 57,614,090 | 120 | $25,830,439.00 | $17,391,279.00 |
| ***2,560.6262*** | *150* | *2,560,626* | *25,606,260* | *51* | *$11,470,961.00* | *$7,391,293.00* |
| ***1,440.3522*** | *200* | *1,440,352* | *14,403,520* | *29* | *$6,886,164.00* | *$4,202,892.00* |
| ***921.8254*** | 250 | 921,825 | 9,218,250 | 18 | $4,597,516.00 | $2,608,692.00 |
| ***640.1565*** | 300 | 640,157 | 6,401,570 | 13 | $3,556,539.00 | $1,884,055.00 |
| ***470.3191*** | 350 | 470,319 | 4,703,190 | 10 | $2,928,953.00 | $1,449,273.00 |
| ***360.0881*** | *400* | *360,088* | *3,600,880* | *8* | *$2,515,563.00* | *$1,159,419.00* |
| *^1^This does not include mosquito dispersal/application costs.* | | | | | | |

# References

Balestrino, F., M. Q. Benedict, and J. R. L. Gilles. 2012. “A New Larval Tray and Rack System for Improved Mosquito Mass Rearing.” *Journal of Medical Entomology* 49 (3): 595–605. <https://doi.org/10.1603/ME11188>.

Benedict, Mark Q., Austin Burt, Margareth L. Capurro, Paul De Barro, Alfred M. Handler, Keith R. Hayes, John M. Marshall, Walter J. Tabachnick, and Zach N. Adelman. 2018. “Recommendations for Laboratory Containment and Management of Gene Drive Systems in Arthropods.” *Vector-Borne and Zoonotic Diseases* 18 (1): 2–13. <https://doi.org/10.1089/vbz.2017.2121>.

Kandul, Nikolay P., Junru Liu, Hector M. Sanchez C, Sean L. Wu, John M. Marshall, and Omar S. Akbari. 2019. “Transforming Insect Population Control with Precision Guided Sterile Males with Demonstration in Flies.” *Nature Communications* 10 (1): 1–12. <https://doi.org/10.1038/s41467-018-07964-7>.

Ovadia, Yaniv, Yoni Halpern, Dilip Krishnan, Josh Livni, Daniel Newburger, Ryan Poplin, Tiantian Zha, and D. Sculley. 2017. “Learning to Count Mosquitoes for the Sterile Insect Technique.” In *Proceedings of the 23rd ACM SIGKDD International Conference on Knowledge Discovery and Data Mining*, 1943–49. KDD ’17. Halifax, NS, Canada: Association for Computing Machinery. <https://doi.org/10.1145/3097983.3098204>.

Qureshi, Alima, Andrew Aldersley, Brian Hollis, Alongkot Ponlawat, and Lauren J. Cator. 2019. “Male Competition and the Evolution of Mating and Life-History Traits in Experimental Populations of *Aedes Aegypti*.” *Proceedings of the Royal Society B: Biological Sciences* 286 (1904): 20190591. <https://doi.org/10.1098/rspb.2019.0591>.

Verdonschot, Piet F. M., and Anna A. Besse-Lototskaya. 2014. “Flight Distance of Mosquitoes (Culicidae): A Metadata Analysis to Support the Management of Barrier Zones Around Rewetted and Newly Constructed Wetlands.” *Limnologica (Online)* 45 (March): 69–79. <https://doi.org/10.1016/j.limno.2013.11.002>.

WHO, World Health. 2020. “WHO | The Mosquito.” *WHO*.

Zhang, Dongjing, Meichun Zhang, Yu Wu, Jeremie R. L. Gilles, Hanano Yamada, Zhongdao Wu, Zhiyong Xi, and Xiaoying Zheng. 2017. “Establishment of a Medium-Scale Mosquito Facility: Optimization of the Larval Mass-Rearing Unit for *Aedes Albopictus* *(Diptera:* *Culicidae)*.” *Parasites & Vectors* 10 (1): 569. <https://doi.org/10.1186/s13071-017-2511-z>.

# Supplemental Materials Section 1: Code used to infer infrastructure costs

Below are two versions of the same code. The first outputs an *.html* document that will be placed in a folder called *MosquitoCosts* on the desktop of your computer. The *MosquitoCosts* folder will be automatically generated by the code. The html document produced by this code should open automatically as a tab on your internet browser. The second version is the same code in the form of a function. The function outputs a table to an Integrated Development Environment (IDE) such as [Rstudio], and can be used in R to enable additional modifications and report development.

## Cost estimator code with HTML table output for *A. aegypti* cost calculator

rm(list = ls())
try(graphics.off(), T)
# For further information about this calculator please contact the author at:
#
# Adam E. Vorsino, Ph.D.
# Strategic Habitat Conservation Division
# Pacific Island Fish and Wildlife Office
# 300 Ala Moana Blvd. Ste. 3-122
# adam_vorsino@fws.gov
# (808)792-9431


# Ecology and Biology
# Verdonschot, Piet F.M., and Anna A. Besse-Lototskaya. âFlight Distance of Mosquitoes (Culicidae):
# A Metadata Analysis to Support the Management of Barrier Zones around Rewetted and Newly
# Constructed Wetlands.â Limnologica 45 (March 2014): 69â79. https://doi.org/10.1016/j.limno.2013.11.002.
# Aedes Specific Parameters
 maxRadius <- seq(50, 400, 50) # max meters Ae can fly from cite (above) and WHO website https://www.who.int/denguecontrol/mosquito/en/
 treatmentArea <- Area # number of square km (from Lincoln Wells DOH)
 MaxCollection <- 181 #Max number collected by BG on HI (from Lincoln Wells DOH)
 MeanCollection <- 15 # Mean number collected by BG on HI (from Lincoln Wells DOH)
 MaxDensity1000km2 <- ((MaxCollection/(pi*maxRadius^2))*1000000) #* treatmentArea # 1000000m = 1 km^2
 MeanDensity1000km2<- ((MeanCollection/(pi*maxRadius^2))*1000000) #* treatmentArea

 Aedes.Mos.Density.km <- MaxDensity1000km2
 Aedes.Mos.Sites <- maxRadius#c('Malama Ki','Nanawale',"Bryson's",'Waiakea','Cooper','Crater',"Pu'u")#,'CJR',"Solomon's")
 Aedes.Elev.m <- maxRadius# c(25, 36, 314, 885, 1024, 1177, 1247)#, 1678, 1686)
 Total.Area.ToCntrl.km <- treatmentArea#24# kilometers squared
 # FemalePercent <- 70 # Sex ratio
 # Overflooding_Multiplier <- 10 # Multiplier to derive the ratio needed for control

 # Operations/Supplies Costs
 Year1.Only.Items <- c('ACL2', 'Iradiator', 'Mosquito.Sex.Sorters (6)', 'larvae Rearing Units (5)',
 'adult cages (100)', 'ovitraps (300)', 'BG traps (50)', 'PCR Machine')#, 'modulare office (60x24ft)')
 Year1.Only.Costs <- c(800000, Irradiator, 6900, 134500, 11040, 2400, 7500, 47000)#, 40000)

 # Office Space <- #, 'modulare office (60x24ft)' can be split with $800/month utilities
 # https://www.willscot.com/mobile-offices/modular-complexes/60-x-24-Modular-Office
 # or
 # https://www.willscot.com/FLEX
 Electricity <- 2000 * 12
 # From Dr. Zhiyong Xi:
 # "Normally I would suggest starting with a space 300-500 m2 and targeting for producing 1 million males per week (see the attached PPT that I used for IAEA meeting last year). It would make too costly and not feasible if the production scale is low (e.g, 100-200k) as you will still need space for adult, larvae rearing, sex separation, irradiator, adult package and quality control. In another word, you may not save too much space with a low production scale. Thus, when you mention “to house 1 rearing unit associated staff etc., a facility would need at minimum a 12x30 foot laboratory space”, I am afraid this space is not enough for you to produce this amount. I think a minimum size of mass rearing facility would need two mobile 24x60 foot laboratories, which should be able to produce 500k to 1 million males per week (or even more if system optimized)..."
 # LaboratorySpace <- 800 * median(c((171000/((72*60)* 0.092903)),
 # (150000/((60*60)* 0.092903)),
 # (180000/((60*48)* 0.092903))))

 # Personel Costs
 Personel.Des <- c('Mass Rearing', 'Quality Control')#, 'Feild Release', 'Community Education')
 Wage.Mass.Rearing <- 15 #15 dollars/hour
 Wage.Quality.Control <- 17 #17 dollars/hour
 HoursPerYear <- 260 * 8 # 260 is 52 weeks/year 5 days/week * 8 hours per day
 # RCUH fringe is set at 61.56% for 2018 see:
 # http://www.ors.hawaii.edu/index.php/apply/budget-development/fringe-benefit-rates
 Fringe <- 0.6156

 # Misc Costs
 AllOtherYear.Items <- 'Misc PCR/Lab/Feild Supplies'
 AllOtherYear.Costs <- 30000 # ensureing that it only changes for every 0.5 increment

 ##################################################################################
 ###########DO NOT MODIFY PAST THIS SECTION########################################
 ##################################################################################

 pckgs <- c('stringr', 'kableExtra', 'Hmisc', 'formattable', 'flextable')

 for(pck in pckgs){
 if ((is.na(installed.packages()[,"Package"][pck]))==T){
 install.packages(pck, dependencies = T)
 }
 library(pck,character.only = T)

 }
 Total.Area.ToCntrl.km <- 1000

 #Bio
 r = 1-(FemalePercent/100) #male proportion given female percent
 wildtypefmales <- 0.5
 WildType.Males.km <- round((((Total.Area.ToCntrl.km*Aedes.Mos.Density.km))/(wildtypefmales)) * 0.5) #(Samuels et al. 2014 numbers are only for females) assumes wildtype equal sex ratio
 # OVerflooding ratio is defined as the ratio of sterile to wild males
 Wolb.Males.Needed.km <- round(WildType.Males.km*Overflooding_Multiplier)

 #Office
 OfficeCosts <- LaboratorySpace + Electricity

 #Personel
 Wage <- c(Wage.Mass.Rearing, Wage.Quality.Control)
 Personel.CossA <- rep(HoursPerYear*Wage)#, length(Personel.Des)) # 260 hours per year
 Personel.Costs <- Personel.CossA # removing 6 positions because community education and feild release
 Fringe.Costs <- Personel.Costs * Fringe
 Tot.Pers.Costs <- (Personel.Costs + Fringe.Costs) * c(8,3)


 #COSTS FOR A Facility 1-300k males
 # (defined by assuming that each 1 mosquito larvae rearing unit can
 # break 300k males)
 # what to rear a min. of 500 - 1 million males...
 # 1/1.5 = 0.6666667 initial cost estimates were for 1.5 million males. This is for ~ 1 million
 # 0.5/1.5 = 0.3333333 this is for ~ 500k males
 Year1.Only.Costs.300k <- Year1.Only.Costs
 Year1.Only.Costs.300k[2:6] <- Year1.Only.Costs[2:6]/5
 OfficeCosts.300k <- OfficeCosts/5 # Assumes 2*24*26 ft containers is min at $120k
 Tot.Pers.Costs.300k <- (Personel.Costs + Fringe.Costs) * (c(8,3)/5)
 AllOtherYear.Costs.300k <- AllOtherYear.Costs/5

 MaxCeiling <- 1000000 * (r)# 1 million Culicidae per rearing unit

 Wolb.Males.Produced <- Wolb.Males.Needed.km * r

 PropWildToLab <- Wolb.Males.Produced/Wolb.Males.Needed.km
 diff.ratio <- ceiling(signif(round(Wolb.Males.Needed.km/MaxCeiling, 2),2))


 # ensureing a min of a million mailes to be reared
 # diff.ratio[which(diff.ratio < round(500000/MaxCeiling))] <- ceiling(500000/MaxCeiling)

 # Defining the numbers for each scenario
 AmountNeeded <- data.frame()
 for(rate in diff.ratio){

 # YEAR 1 COSTS
 Yr1.ItemCostJnk <- Year1.Only.Costs.300k
 Yr1.ItemCostJnk[2:6] <- Year1.Only.Costs.300k[2:6] * rate
 # PCR machine is for each 1.5 million so
 rate2 <- ceiling(rate/5)
 Yr1.ItemCostJnk[7] <- Year1.Only.Costs.300k[7] * rate2

 Yr1.PersCostJnk <- (Personel.Costs + Fringe.Costs) * ((c(8,3)/5)*rate)
 Yr1.MiscJnk <- AllOtherYear.Costs.300k*rate

 rate3 <- rate#ceiling(rate/2.5)
 Yr1.OfficeCostsJnk <- OfficeCosts.300k * rate3
 Yr1.TotalCosts <- currency(sum(Yr1.ItemCostJnk, Yr1.PersCostJnk, Yr1.MiscJnk, Yr1.OfficeCostsJnk))


 # SUBSEQUENT YEAR COSTS
 SUB.PersCostJnk <- (Personel.Costs + Fringe.Costs) * ((c(8,3)/5)*rate)
 SUB.MiscJnk <- AllOtherYear.Costs.300k*rate
 SUB.OfficeCostsJnk <- Electricity * rate3
 SUB.TotalCosts <- currency(sum(SUB.PersCostJnk, SUB.MiscJnk, SUB.OfficeCostsJnk))

 AmountNeeded <- rbind(AmountNeeded, cbind(rate, round(Yr1.TotalCosts), round(SUB.TotalCosts)))#Total.Area.ToCntrl.km,

 }
 AmountNeeded$V3 <- currency(AmountNeeded$V3)
 AmountNeeded$V2 <- currency(AmountNeeded$V2)


 AmountNeeded2 <- data.frame(cbind(Aedes.Mos.Density.km, Aedes.Mos.Sites, WildType.Males.km, Wolb.Males.Needed.km, AmountNeeded))#
 # Aedes.Elev.m,
 colnames(AmountNeeded2) <- capitalize(c('Culicid Density per Km2','Flight Distance meters', 'Wild Type Males', 'IIT:Wild Type Males (10:1)',
 'Rate Used', 'First Year Cost ($)', 'Subsequent Year Costs ($)'))

for(tocomma in 2:4){
 AmountNeeded2[,tocomma] <- comma(AmountNeeded2[, tocomma], digits = 0L)
}


# Partitions <- c(5,7)
#'Area of Control (sqr. km.)',
PrCntrlTable <- kable(AmountNeeded2, #title = "<b>IIT/SIT Male <i>Aedes aegypti</i> Mass Release Production and Costs",
 caption = paste0('<b>IIT/SIT Male <i>Aedes aegypti</i> Mass Release Production and Costs for a ',
 Total.Area.ToCntrl.km,
 ' km<sup>2</sup> area. In the table a rate of 1 is equivalent to the production of &le; 500,000 IIT/SIT males.
 This does NOT include costs associated with community outreach or field releases. Rows higlighted in red denote
 possible flight distances derived from Verdonschot <i>et al.</i> (2014), and those in blue represent the average distance
 <i>Aedes aegypti</i> adults fly in a lifetime, derived from the World Health Organization (WHO) estimate.</b>'),
 escape = F, format = 'html') %>%
 kable_styling("striped", full_width = F) %>%
 # group_rows(toupper(paste0("Low Elevation")), 1, Partitions[1]-1, indent = F) %>%
 # group_rows(toupper(paste0("Mid Elevation")), Partitions[1], Partitions[2], indent = F) %>%
 row_spec(row = 0, bold = T, align = 'center') %>%
 row_spec(row = 1:nrow(AmountNeeded2), align = 'center') %>%
 row_spec(3:4, bold = T, color = "white", background = "darkred") %>%
row_spec(8, bold = T, color = "white", background = "darkblue")%>%
 add_footnote(c('Note: Verdonschot, Piet F.M., and Anna A. Besse-Lototskaya. Limnologica 45 (March 2014): 69â79. https://doi.org/10.1016/j.limno.2013.11.002.',
 'Note: World Health Organization (WHO) flight distance information (accessed 4/18/2020): https://www.who.int/denguecontrol/mosquito/en/',
 'Note: This does not include mosquito dispersal/application costs.'), notation = 'number')

#
#
kableExtra:::as_image(PrCntrlTable, file = paste0(WorkFolder, 'Rearing Costs for ',
 Total.Area.ToCntrl.km , 'sqr.km_1.html'))
#

# as_image(PrCntrlTable, file = paste0(WorkFolder, 'Rearing Costs for ',
# Total.Area.ToCntrl.km , 'sqr.km_2.pdf'))rm(list = ls())


 # caption = paste0('<b>IIT/SIT Male <i>Aedes aegypti</i> Mass Release Production and Costs for a ',
 # Total.Area.ToCntrl.km,
 # ' km<sup>2</sup> area. In the table a rate of 1 is equivalent to the production of &le; 500,000 IIT/SIT males.
 # This does NOT include costs associated with community outreach or field releases. Rows higlighted in red denote
 # possible flight distances derived from Verdonschot <i>et al.</i> (2014), and those in blue represent the average distance
 # <i>Aedes aegypti</i> adults fly in a lifetime, derived from the World Health Organization (WHO) estimate.</b>')
 # add_footnote(c('Note: Verdonschot, Piet F.M., and Anna A. Besse-Lototskaya. Limnologica 45 (March 2014): 69â79. https://doi.org/10.1016/j.limno.2013.11.002.',
 # 'Note: World Health Organization (WHO) flight distance information (accessed 4/18/2020): https://www.who.int/denguecontrol/mosquito/en/',
 # 'Note: This does not include mosquito dispersal/application costs.'), notation = 'number')

## Cost estimator function with table output for *A. aegypti* cost calculator

# For further information about this calculator please contact the author at:
#
# Adam E. Vorsino, Ph.D.
# Strategic Habitat Conservation Division
# Pacific Island Fish and Wildlife Office
# 300 Ala Moana Blvd. Ste. 3-122
# adam_vorsino@fws.gov
# (808)792-9431
LaboratorySpaceA <- 800 * median(c((171000/((72*60)* 0.092903)),
 (150000/((60*60)* 0.092903)),
 (180000/((60*48)* 0.092903))))
IrradiatorA <- 250000
Aed_CostEst <- function(FemalePercent, Overflooding_Multiplier, Area, LaboratorySpace, Irradiator = IrradiatorA){

 # # Verdonschot, Piet F.M., and Anna A. Besse-Lototskaya. âFlight Distance of Mosquitoes (Culicidae):
# # A Metadata Analysis to Support the Management of Barrier Zones around Rewetted and Newly
# # Constructed Wetlands.â Limnologica 45 (March 2014): 69â79. https://doi.org/10.1016/j.limno.2013.11.002.
 maxRadius <- seq(50, 400, 50) # max meters Ae can fly from cite (above) and WHO website https://www.who.int/denguecontrol/mosquito/en/
 treatmentArea <- Area # number of square km (from Lincoln Wells DOH)
 MaxCollection <- 181 #Max number collected by BG on HI (from Lincoln Wells DOH)
 MeanCollection <- 15 # Mean number collected by BG on HI (from Lincoln Wells DOH)
 MaxDensity1000km2 <- ((MaxCollection/(pi*maxRadius^2))*1000000) #* treatmentArea # 1000000m = 1 km^2
 MeanDensity1000km2<- ((MeanCollection/(pi*maxRadius^2))*1000000) #* treatmentArea

 Aedes.Mos.Density.km <- MaxDensity1000km2
 Aedes.Mos.Sites <- maxRadius#c('Malama Ki','Nanawale',"Bryson's",'Waiakea','Cooper','Crater',"Pu'u")#,'CJR',"Solomon's")
 Aedes.Elev.m <- maxRadius# c(25, 36, 314, 885, 1024, 1177, 1247)#, 1678, 1686)
 Total.Area.ToCntrl.km <- treatmentArea#24# kilometers squared
 # FemalePercent <- 70 # Sex ratio
 # Overflooding_Multiplier <- 10 # Multiplier to derive the ratio needed for control

 # Operations/Supplies Costs
 Year1.Only.Items <- c('ACL2', 'Iradiator', 'Mosquito.Sex.Sorters (6)', 'larvae Rearing Units (5)',
 'adult cages (100)', 'ovitraps (300)', 'BG traps (50)', 'PCR Machine')#, 'modulare office (60x24ft)')
 Year1.Only.Costs <- c(800000, Irradiator, 6900, 134500, 11040, 2400, 7500, 47000)#, 40000)

 # Office Space <- #, 'modulare office (60x24ft)' can be split with $800/month utilities
 # https://www.willscot.com/mobile-offices/modular-complexes/60-x-24-Modular-Office
 # or
 # https://www.willscot.com/FLEX
 Electricity <- 2000 * 12
 # From Dr. Zhiyong Xi:
 # "Normally I would suggest starting with a space 300-500 m2 and targeting for producing 1 million males per week (see the attached PPT that I used for IAEA meeting last year). It would make too costly and not feasible if the production scale is low (e.g, 100-200k) as you will still need space for adult, larvae rearing, sex separation, irradiator, adult package and quality control. In another word, you may not save too much space with a low production scale. Thus, when you mention “to house 1 rearing unit associated staff etc., a facility would need at minimum a 12x30 foot laboratory space”, I am afraid this space is not enough for you to produce this amount. I think a minimum size of mass rearing facility would need two mobile 24x60 foot laboratories, which should be able to produce 500k to 1 million males per week (or even more if system optimized)..."
 # LaboratorySpace <- 800 * median(c((171000/((72*60)* 0.092903)),
 # (150000/((60*60)* 0.092903)),
 # (180000/((60*48)* 0.092903))))

 # Personel Costs
 Personel.Des <- c('Mass Rearing', 'Quality Control')#, 'Feild Release', 'Community Education')
 Wage.Mass.Rearing <- 15 #15 dollars/hour
 Wage.Quality.Control <- 17 #17 dollars/hour
 HoursPerYear <- 260 * 8 # 260 is 52 weeks/year 5 days/week * 8 hours per day
 # RCUH fringe is set at 61.56% for 2018 see:
 # http://www.ors.hawaii.edu/index.php/apply/budget-development/fringe-benefit-rates
 Fringe <- 0.6156

 # Misc Costs
 AllOtherYear.Items <- 'Misc PCR/Lab/Feild Supplies'
 AllOtherYear.Costs <- 30000 # ensureing that it only changes for every 0.5 increment

 ##################################################################################
 ###########DO NOT MODIFY PAST THIS SECTION########################################
 ##################################################################################

 pckgs <- c('stringr', 'kableExtra', 'Hmisc', 'formattable', 'flextable')

 for(pck in pckgs){
 if ((is.na(installed.packages()[,"Package"][pck]))==T){
 install.packages(pck, dependencies = T)
 }
 library(pck,character.only = T)

 }
 Total.Area.ToCntrl.km <- 1000

 #Bio
 r = 1-(FemalePercent/100) #male proportion given female percent
 wildtypefmales <- 0.5
 WildType.Males.km <- round((((Total.Area.ToCntrl.km*Aedes.Mos.Density.km))/(wildtypefmales)) * 0.5) #(Samuels et al. 2014 numbers are only for females) assumes wildtype equal sex ratio
 # OVerflooding ratio is defined as the ratio of sterile to wild males
 Wolb.Males.Needed.km <- round(WildType.Males.km*Overflooding_Multiplier)

 #Office
 OfficeCosts <- LaboratorySpace + Electricity

 #Personel
 Wage <- c(Wage.Mass.Rearing, Wage.Quality.Control)
 Personel.CossA <- rep(HoursPerYear*Wage)#, length(Personel.Des)) # 260 hours per year
 Personel.Costs <- Personel.CossA # removing 6 positions because community education and feild release
 Fringe.Costs <- Personel.Costs * Fringe
 Tot.Pers.Costs <- (Personel.Costs + Fringe.Costs) * c(8,3)


 #COSTS FOR A Facility 1-300k males
 # (defined by assuming that each 1 mosquito larvae rearing unit can
 # break 300k males)
 # what to rear a min. of 500 - 1 million males...
 # 1/1.5 = 0.6666667 initial cost estimates were for 1.5 million males. This is for ~ 1 million
 # 0.5/1.5 = 0.3333333 this is for ~ 500k males
 Year1.Only.Costs.300k <- Year1.Only.Costs
 Year1.Only.Costs.300k[2:6] <- Year1.Only.Costs[2:6]/5
 OfficeCosts.300k <- OfficeCosts/5 # Assumes 2*24*26 ft containers is min at $120k
 Tot.Pers.Costs.300k <- (Personel.Costs + Fringe.Costs) * (c(8,3)/5)
 AllOtherYear.Costs.300k <- AllOtherYear.Costs/5

 MaxCeiling <- 1000000 * (r)# 1 million Culicidae per rearing unit

 Wolb.Males.Produced <- Wolb.Males.Needed.km * r

 PropWildToLab <- Wolb.Males.Produced/Wolb.Males.Needed.km
 diff.ratio <- ceiling(signif(round(Wolb.Males.Needed.km/MaxCeiling, 2),2))


 # ensureing a min of a million mailes to be reared
 # diff.ratio[which(diff.ratio < round(500000/MaxCeiling))] <- ceiling(500000/MaxCeiling)

 # Defining the numbers for each scenario
 AmountNeeded <- data.frame()
 for(rate in diff.ratio){

 # YEAR 1 COSTS
 Yr1.ItemCostJnk <- Year1.Only.Costs.300k
 Yr1.ItemCostJnk[2:6] <- Year1.Only.Costs.300k[2:6] * rate
 # PCR machine is for each 1.5 million so
 rate2 <- ceiling(rate/5)
 Yr1.ItemCostJnk[7] <- Year1.Only.Costs.300k[7] * rate2

 Yr1.PersCostJnk <- (Personel.Costs + Fringe.Costs) * ((c(8,3)/5)*rate)
 Yr1.MiscJnk <- AllOtherYear.Costs.300k*rate

 rate3 <- rate#ceiling(rate/2.5)
 Yr1.OfficeCostsJnk <- OfficeCosts.300k * rate3
 Yr1.TotalCosts <- currency(sum(Yr1.ItemCostJnk, Yr1.PersCostJnk, Yr1.MiscJnk, Yr1.OfficeCostsJnk))


 # SUBSEQUENT YEAR COSTS
 SUB.PersCostJnk <- (Personel.Costs + Fringe.Costs) * ((c(8,3)/5)*rate)
 SUB.MiscJnk <- AllOtherYear.Costs.300k*rate
 SUB.OfficeCostsJnk <- Electricity * rate3
 SUB.TotalCosts <- currency(sum(SUB.PersCostJnk, SUB.MiscJnk, SUB.OfficeCostsJnk))

 AmountNeeded <- rbind(AmountNeeded, cbind(rate, round(Yr1.TotalCosts), round(SUB.TotalCosts)))#Total.Area.ToCntrl.km,

 }
 AmountNeeded$V3 <- currency(AmountNeeded$V3)
 AmountNeeded$V2 <- currency(AmountNeeded$V2)


 AmountNeeded2 <- data.frame(cbind(Aedes.Mos.Density.km, Aedes.Mos.Sites, WildType.Males.km, Wolb.Males.Needed.km, AmountNeeded))#
 # Aedes.Elev.m,
 colnames(AmountNeeded2) <- capitalize(c('Culicid Density per Km2','Flight Distance meters', 'Wild Type Males', 'IIT:Wild Type Males (10:1)',
 'Rate Used', 'First Year Cost ($)', 'Subsequent Year Costs ($)'))


 Partitions <- c(5,7)
 AmountNeeded3 <- AmountNeeded2
 PrCntrlTable <- flextable(data.frame(AmountNeeded3)) %>%
 color(i = c(3:4), color="darkred", part = 'body') %>%
 italic(i = c(3:4), italic = TRUE, part = "body") %>%
 color(i = c(8), color="darkblue", part = 'body') %>%
 italic(i = c(8), italic = TRUE, part = "body") %>%
 italic(j = 1, italic = TRUE, part = "body") %>%
 bold(j = 1, bold = TRUE, part = "body") %>%
 set_header_labels(Culicid.Density.per.Km2 = 'Culicid Density (per square km.)',
 Flight.Distance.meters = 'Flight Distance (meters)',
 Wild.Type.Males = 'Wild Type Males',
 IIT.Wild.Type.Males..10.1. = 'IIT:Wild Type Males (10:1)',
 Rate.Used = 'Rate Used',
 First.Year.Cost.... = 'First Year Cost ($)',
 Subsequent.Year.Costs.... = 'Subsequent Year Costs ($)') %>%

 theme_zebra(odd_header="transparent") %>%
 align(align = "center", part = "header") %>%
 valign(valign = "center", part = "all") %>%
 # hline(i=c(1,5:6), part = "body", border = officer::fp_border()) %>%
 # hline(i=c(1), part = "header", border = officer::fp_border()) %>%
 hline(i=c(2,4), part = "body", border = officer::fp_border(width = 3, color = 'darkred')) %>%
 hline(i=c(7:8), part = "body", border = officer::fp_border(width = 3, color = 'darkblue')) %>%
 hline(i=1, part = "header", border = officer::fp_border()) %>%
 vline(j=1, part = "body", border = officer::fp_border()) %>%
 fontsize(i = 1, part = "header", size = 12) %>%
 align(align = 'center', part = 'all') %>%
 # align(d'da) %>%
 colformat_int(j = 2:7, big.mark = ",") %>%
 autofit() %>%
 fontsize(size = 12, part = 'body') %>%
 fit_to_width(max_width = 7.25) %>%
 # set_caption(paste0('IIT/SIT Male **Aedes aegypti** Mass Release Production and Costs for a ', Total.Area.ToCntrl.km, ' km^2^ area. In the table a rate of 1 is equivalent to the production of \u2264 1 million IIT/SIT Culicidae using the default 0.3:1 female biased sex ratio. Rows higlighted in red denote possible flight distances derived from Verdonschot *et al* (2014), and those in blue represent the average distance **A. aegypti** adults fly in a lifetime, derived from the World Health Organization (WHO) estimate.')) %>%
 footnote(value = as_paragraph('This does not include mosquito dispersal/application costs.'), part = 'header', i = 1, j = c(6),
 ref_symbols = c('1')) %>%
 fontsize(part = 'footer', size = 8) %>%
 italic(part = 'footer', italic = T)#,

 # add_footnote(c('Note: Verdonschot, Piet F.M., and Anna A. Besse-Lototskaya. Limnologica 45 (March 2014): 69â79. https://doi.org/10.1016/j.limno.2013.11.002.',
 # 'Note: World Health Organization (WHO) flight distance information (accessed 4/18/2020): https://www.who.int/denguecontrol/mosquito/en/',
 # 'Note: This does not include mosquito dispersal/application costs.'), notation = 'number')

 PrCntrlTable
}
Aed_CostEst(FemalePercent = 70, Overflooding_Multiplier = 10, Area = 1000, LaboratorySpace = LaboratorySpaceA)
